# Lifetime evaluation of left ventricular structure and function in male ApoE null mice after gamma and space-type radiation exposure

**DOI:** 10.3389/fphys.2023.1292033

**Published:** 2023-11-20

**Authors:** Agnieszka Brojakowska, Cedric J. Jackson, Malik Bisserier, Mary K. Khlgatian, Vineeta Jagana, Abrisham Eskandari, Cynthia Grano, Steve R. Blattnig, Shihong Zhang, Kenneth M. Fish, Vadim Chepurko, Elena Chepurko, Virginia Gillespie, Ying Dai, Amit Kumar Rai, Venkata Naga Srikanth Garikipati, Lahouaria Hadri, Raj Kishore, David A. Goukassian

**Affiliations:** ^1^ Cardiovascular Research Institute, Icahn School of Medicine at Mount Sinai, New York, NY, United States; ^2^ Yale School of Medicine, New Haven, CT, United States; ^3^ National Institute of Aerospace, Hampton, VA, United States; ^4^ New York Medical College, Valhalla, New York, United States; ^5^ National Aeronautics and Space Administration, Hampton, VA, United States; ^6^ Center for Comparative Medicine and Surgery, Icahn School of Medicine at Mount Sinai, New York, NY, United States; ^7^ Aging and Cardiovascular Discovery Center, Lewis Katz School of Medicine, Temple University, Philadelphia, PA, United States; ^8^ Center of Excellence for Translational Medicine and Pharmacology/Department of Pharmacological Sciences, Icahn School of Medicine at Mount Sinai, New York, NY, United States; ^9^ Department of Cardiovascular Sciences, Center for Translational Medicine, Lewis Katz School of Medicine, Temple University, Philadelphia, PA, United States

**Keywords:** ionizing space radiation, cardiovascular disease, echocardiography, mathematical modeling, atherosclerosis

## Abstract

The space radiation (IR) environment contains high charge and energy (HZE) nuclei emitted from galactic cosmic rays with the ability to overcome current shielding strategies, posing increased IR-induced cardiovascular disease risks for astronauts on prolonged space missions. Little is known about the effect of 5-ion simplified galactic cosmic ray simulation (simGCRsim) exposure on left ventricular (LV) function. Three-month-old, age-matched male Apolipoprotein E (ApoE) null mice were irradiated with ^137^Cs gamma (γ; 100, 200, and 400 cGy) and simGCRsim (50, 100, 150 cGy all at 500 MeV/nucleon (n)). LV function was assessed using transthoracic echocardiography at early/acute (14 and 28 days) and late/degenerative (365, 440, and 660 days) times post-irradiation. As early as 14 and 28-days post IR, LV systolic function was reduced in both IR groups across all doses. At 14 days post-IR, 150 cGy simGCRsim-IR mice had decreased diastolic wall strain (DWS), suggesting increased myocardial stiffness. This was also observed later in 100 cGy γ-IR mice at 28 days. At later stages, a significant decrease in LV systolic function was observed in the 400 cGy γ-IR mice. Otherwise, there was no difference in the LV systolic function or structure at the remaining time points across the IR groups. We evaluated the expression of genes involved in hemodynamic stress, cardiac remodeling, inflammation, and calcium handling in LVs harvested 28 days post-IR. At 28 days post-IR, there is increased expression of *Bnp* and *Ncx* in both IR groups at the lowest doses, suggesting impaired function contributes to hemodynamic stress and altered calcium handling. The expression of *Gals3* and *β-Mhc* were increased in simGCRsim and γ-IR mice respectively, suggesting there may be IR-specific cardiac remodeling. IR groups were modeled to calculate the Relative Biological Effectiveness (RBE) and Radiation Effects Ratio (RER). No lower threshold was determined using the observed dose-response curves. These findings do not exclude the possibility of the existence of a lower IR threshold or the presence of IR-induced cardiovascular disease (CVD) when combined with additional space travel stressors, e.g., microgravity.

## Introduction

The hazardous space environment brings many concerns of acute and prolonged exposure to space environment stressors for human health including but not limited to microgravity and radiation. Considerations for the acute- and long-term degenerative effects of space travel on health remain emergent with upcoming expeditions to the Moon and Mars. In particular, the effects of space radiation (IR) on cardiovascular disease (CVD) risk are of particular concern and relevant to the National Aeronautics and Space Administration (NASA) during exploration-type space missions. Radiation-induced CVD (RICVD) is a concern following low-linear energy transfer (LET) radiation, such as gamma (γ)-IR, in patients undergoing radiation therapy (RT) for thoracic malignancies, as well as industrial and occupational exposures, and in A-bomb survivors (Reinders et al., 1999; Berrington et al., 2001; National Library of Medicine, 2002; Grube et al., 2003; Heidenreich et al., 2003; Li et al., 2009; Andreassi et al., 2015). Follow-up studies of patients receiving RT in the vicinity of the heart have an increased risk of developing accelerated coronary artery disease, cardiomyopathies, myocarditis, pericardial disease, supraventricular arrhythmias, and valvular disease (Hoon et al., 2007; Weintraub et al., 2010; Nielsen et al., 2017; Wang et al., 2020; Koutroumpakis et al., 2022). Notably, while certain inflammatory processes (e.g., myocarditis or pericarditis) may occur acutely, most CVDs present months to decades after initial exposure. NASA’s predominant risk estimates are modeled with respect to these terrestrial radiation exposures, which limits the recapitulation of the quality, dose and dose-rate of radiation astronauts will be exposed to throughout deep-space missions and their adverse health effects.

The space IR environment includes various charged particles resulting from galactic cosmic rays (GCRs), solar emissions in the form of solar energetic particles (SEPs), and trapped radiation in the Van Allen’s belt around the Earth. Unlike GCRs, SEPs are predominantly composed of low/medium energy protons (^1^H), which can be effectively shielded. Exposure to GCRs has become increasingly concerning for missions beyond the Low Earth Orbit (LEO), where the protective magnetosphere cannot provide additional shielding. GCRs are fully ionized atomic nuclei originating beyond the solar system and contain high-energy ^1^H (90%), helium particles (9%), and HZE [high charge (H), atomic number (Z), and energy (E)] nuclei (1%) (Nelson, 2016). In particular, HZE nuclei have high LET, which penetrate spacecraft shielding and produce complex dense ionizing tracks that can generate secondary refractory particles that introduce clustered damage to traversed and neighboring cells (Barcellos-Hoff and Mao, 2016). While increasing studies have investigated singular HZE nuclei effects on CVDs, the acute and long-term effects of complex HZE exposure (i.e., multiple heavy ions) on CVD risk remain largely unknown.

There is a paucity of relevant human data to estimate space IR-induced CVD risk. Over the last decade, an increasing number of studies on HZE exposure using animal models have begun to address the gaps in space IR-induced CVD risks for astronauts in deep-space missions. Our team has previously shown that following whole-body irradiation with 0.15 Gy iron [^56^Fe; 1 GeV/nucleon (n)], male C57Bl/6NT exhibit a significant decline in cardiac function detected by decreased dP/dt_max_ and dP/dt_min_ (dP/dt_max_ - maximum derivative of change in systolic pressure over time and dP/dt_min_ - minimum derivative of change in diastolic pressure over time) and increased LV end-diastolic pressure at 1 and 3 months post IR, but later see recovery in function at 10 months (Yan et al., 2014). In contrast, male C57Bl/6NT irradiated with 0.5 Gy ^1^H (1 GeV) show preserved cardiac function with progressive cardiac hypertrophy (increased LV posterior wall thickness and NFATc4 expression, an indicator of increased cardiac hypertrophy signaling) at 1-month post IR followed by a decline in cardiac function at 10 months. This suggested the possibility of IR-specific effects on cardiac function and remodeling. Seawright et al. showed that male C56BL/6J mice exposed to oxygen ions (^16^O, 0.05–1 Gy, 600 MeV/n) showed decreased LV systolic function at 3- and 7-months post IR with evidence of cardiac remodeling (increased collagen deposition and α-smooth muscle actin-αSMA deposition). These effects did not differ when animals were initially irradiated with ^1^H (0.5 Gy, 150 MeV) (Se et al., 2019). Interestingly, a study of C57BL/6J male mice initially irradiated with ^1^H (0.1 Gy, 150 MeV) followed by ^56^Fe (0.5 Gy, 600 MeV/n) showed a priming effect, with a decrease in the expression of αSMA, type III collagen, and other inflammatory markers, which are elevated when the mice were irradiated solely with ^56^Fe ions (Ramadan et al., 2016). Considering the differential effects on cardiac function and remodeling noted based on HZE type, dose, duration, and sequence of exposure, further studies are needed to better recapitulate the complexity of space-type IR using 5-ion simGCRsim.

Transthoracic echocardiography is a non-invasive method to assess changes in cardiac function and structure (Gardin et al., 1995). Recently, we showed that wild-type C57BL/6J male mice exposed to various doses of simGCRsim (space-type) or γ-IR displayed an acute decline in LV systolic function as early as 14 and 28 days post-IR, which persisted for 365 days in both the IR groups (Brojakowska et al., 2023). Interestingly, divergent effects were observed 660 days post-IR, when γ-IR but not simGCRsim-IR mice exhibited reduced LV systolic function. In contrast, at 660 days, simGCRsim-IR mice had preserved LV systolic function, which is associated with increased expression of markers of cardiac fibrosis, hypertrophy, and inflammation, suggesting that space IR may induce different remodeling processes predominantly associated with diastolic dysfunction, while systolic function is preserved. We extended this study to investigate the longitudinal effects of γ-IR (100, 200, and 400 cGy) and simGCRsim (50, 100, and 150 cGy/500 MeV/n)-IR on LV structure and function in Apolipoprotein E (ApoE) null male mice. ApoE-null mice are genetically predisposed to atherosclerosis and develop both spontaneous and diet-induced atherosclerotic plaques (Nakashima et al., 1994; van Dijk et al., 1999; Lo Sasso et al., 2016), enabling comparisons to pathologic atherogenesis observed in humans. Here, 3-month-old age-matched ApoE null male mice were exposed to varying doses of γ- and simGCRsim-IR, and acute (14, 28 days post-IR) and long-term degenerative effects (365-, 440-, and 660-days post-IR) on LV function were assessed using echocardiography. This study aimed to: (Li et al., 2009): determine the effects of γ- and simGCRsim-IR on LV function in atherosclerosis-prone mice; (National Library of Medicine, 2002); identify the disease spectrum and latency following single, whole-body IR exposure; (Reinders et al., 1999); determine whether dose thresholds for CVD endpoints exist; and (Heidenreich et al., 2003) describe the comparative impact of γ- and simGCRsim-IR by calculating the Relative Biological Effectiveness (RBE) or the Radiation Effects Ratio (RER).

## Materials and methods

***Animal Procedures.*** All animal procedures were performed in accordance with the standards of the Guide for the Care and Use of Laboratory Animals for the National Institutes of Health and approved by the Animal Care and Use Committees at Brookhaven National Laboratory (BNL) (Upton, NY, United States of America) (BNL IACUC Protocol #502) and the Icahn School of Medicine at Mount Sinai (New York, NY, United States of America) (ISMMS IACUC Protocol #2019-0017).

**FIGURE 1:**
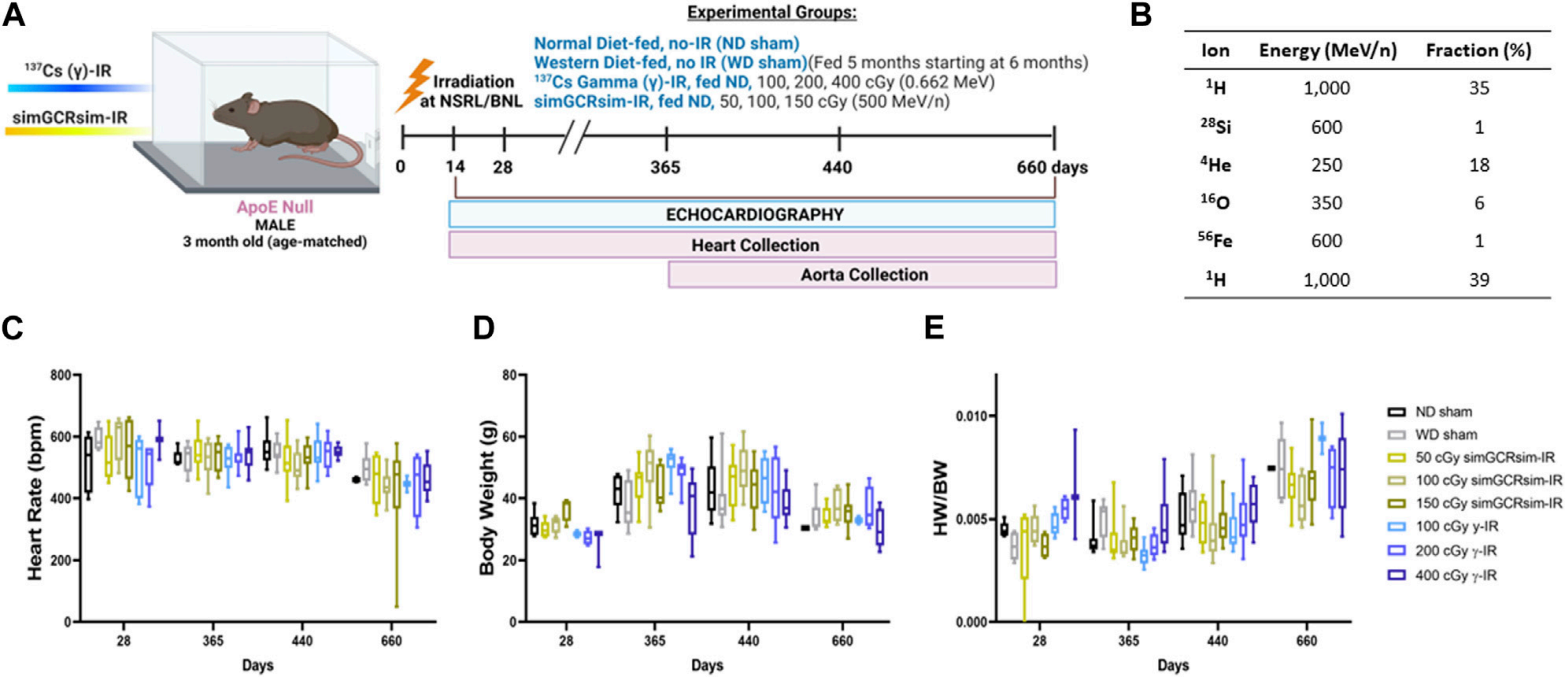
Physiological response of ApoE null male mice to gamma gamma(γ) - and simGCRsim-IR. **(A)** 3-month-old age-matched ApoE Null mice were irradiated with ^137^Cs-γ-IR (100, 200, 400 cGy) and simGCRsim (50, 100, 150 cGy). We assessed LV function by transthoracic echocardiography at 14, 28-, 365-, 440-, and 660-days post-IR. No-IR normal diet (ND)-fed mice served as negative control (ND sham) and no-IR western diet (WD) fed mice served as positive control (WD sham). In addition to transthoracic echocardiography, hearts were collected at each time point. **(B)** Heart rates were obtained during transthoracic echocardiography while mice were anesthetized using isoflurane (3%–4% induction, 1%–2% maintenance). **(C)** Animal Heart rates. **(D)** Animal bodyweights. **(E)** Heart weight to body weight ratio. For acute time points (14 and 28 days), *N* = 3-9 animals (14 days: ND sham N = 9, 50 cGy simGCRsim and 100, 400 cGy γ *N* = 3, 100 cGy simGCRsim *N* = 4, 150 cGy simGCRsim and 200 cGy γ-IR *N* = 5) (28 days: N = 5 except 400 cGy γ N = 3). For the degenerative time points *N* = 7–10 animals for 365 and 440 days, and for 660 days *N = 2* for ND sham and 100 cGy γ-IR, while all other remaining treatment conditions *N = 4–10*. *p-*values were calculated using one-way ANOVA. Due to technical reasons, heart weights were not collected at the 14-day time point.

Of note, all animal experiments, treatments (with exception of added irradiation doses) and modeling were performed as described in our prior study focused on wild-type C57BL/6J male mice and have been adapted and added in this manuscript for clarity and completeness (Brojakowska et al., 2023). Seven hundred and twenty male ApoE null mice (Jackson Laboratory, Bar Harbor, ME, United States) were shipped directly to the Brookhaven Laboratory Animal Facility (BLAF) at Brookhaven National Laboratory (BNL, Brookhaven, NY, United States) approximately 1 week prior to irradiation. Mice remained housed at BLAF for 22 months post-irradiation to reduce the confounding effect of stress associated with additional transportation and required 3-month quarantine at a third-party facility. Animals were housed in groups of 4-5 per cage and provided with water and food *ad libitum*. Both control (non-irradiated) and irradiated mice were fed commercial food chow, referred to as a normal diet (ND). A subset of non-irradiated mice was fed a high-fat Western diet (WD, Teklad TD.88137, Envigo, Indianapolis, IL, United States of America) starting at 6 months of age for a 5-month period, serving as a positive control for ND sham irradiated mice. The experimental design is illustrated in Figure 1A and the number of mice at the start of the experiment for each treatment group/collection time point is presented in Supplementary Table S1. Animals were housed under a 12:12 h (h) light-dark cycle at 20°C–22°C and 30%–70% relative humidity.

Animals were monitored at least once a day for any physical or behavioral changes indicative of distress, discomfort, pain, or injury. Mice displaying general criteria of poor health (e.g., abnormal discharge from orifices, increased respiratory or heart rate, wound dehiscence, neoplasm, weight loss greater than 15%) were euthanized using 100% CO_2_ at a rate of 20% air replacement per minute, followed by neck dislocation. Animals that died spontaneously were grossly examined and frozen until processed for autopsy by a pathologist blindfolded to the study treatment conditions. Multiple organs (heart, lungs, liver, spleen, and kidneys) were collected, formalin-fixed, and examined *postmortem*. The remaining mice were sacrificed via exsanguination at dedicated times post irradiation (14, 28, 365, 440, 660 days) and hearts were collected for qPCR and histological analysis.

***Irradiation Procedures.*** Three-month-old, age-matched male ApoE Null mice were exposed to charged heavy-ion particle beams at the NASA Space Radiation Laboratory (NSRL) and gamma irradiation using ^137^Cesium (^137^Cs) source at the Biology Department at BNL. Dosimetry studies (depth-dose and dose-uniformity measurements) were conducted by beamline physicists. Mice were irradiated with a single full-body dose of ^137^Cs (γ) rays at doses of either 100, 200, or 400 cGy (0.662 MeV), or the simplified 5 ion simGCRsim at doses of 50, 100, or 150 cGy (Simonsen et al., 2020). Delivery of simGCRsim requires approximately 20 min to deliver 6 beams, sequentially. The composition of simGCRsim irradiation, in addition to the order of ion delivery, is shown in Figure 1B. During the time of radiation exposure, unanesthetized mice were held in 127 × 76.2 × 50.8 mm plastic boxes with 20 holes (3 mm in diameter) to provide airflow. Control sham-treated animals were placed in similar boxes for equivalent times but were not irradiated. All γ-ray exposures were conducted at BNL, which has a^137^Cs radiation facility suitable for the low LET γ exposures required to properly calculate the RBEs. The radiation ions, doses, and energies used in these studies were recommended by the Space Radiation Element of NASA’s Human Research Program (HRP).

***Echocardiography.*** Transthoracic echocardiography was performed to assess cardiac structure and function using a GE Vivid E9 with XDclear Cardiac Ultrasound (General Electric Company, Boston, MA, United States) and a GE model i13L probe (General Electric Company, Boston, MA, United States). Echocardiography assessments were conducted by a highly trained technician who was blinded to the treatment groups. The operator was not informed about the specific treatment conditions, such as sham vs. ionizing radiation (IR), or the type of diet (WD/ND) administered to the mice. Blinding was maintained throughout all echocardiographic data acquisition procedures to minimize potential bias and ensure data integrity. Mice were anesthetized with isoflurane (Baxter Healthcare Corporation, Deerfield, IL, United States), induced at 3% and maintained with 1%–2% isoflurane. To optimize imaging, hair was removed from the neckline to the mid-chest (using Nair). Throughout imaging, mice were placed in a supine position on a heated table to maintain a core temperature of 37°C. B- and M-mode images were acquired from the parasternal short-axis view. Parameters assessed during image acquisition were related to overall LV systolic function and cardiac output, including LV ejection fraction (LVEF), LV fractional shortening (LVFS), end-systolic volume (ESV), and end-diastolic volume (EDV). Structural changes were assessed by calculating the dimensions of the LV chamber and respective walls: LV end-diastolic diameter (LVEDD), LV end-systolic diameter (LVESD), posterior wall thickness at end-diastole (LVPWd) and systole (LVPWs), intraventricular septal thickness (IVSd), left ventricular internal diameter at end-diastole (LVIDd), LVID at end systole (LVIDs), and posterior wall thickness in systole and diastole (LVPWs and LVPWd). Additional assessment of any structural or functional attenuation was performed using calculations for LV mass (1.053 × ((LVIDd + LVPWd + IVSd)^3^—LVIDd^3^)), diastolic wall strain (DWS); [IVSd + LVPWd]/LVIDd), and relative wall thickness (RWT; ((2′VPWd)/LVIDd)). Each parameter is reported as the average of three individual measurements (not repeated measures).

***Total RNA Isolation and RT-qPCR Analysis.*** Total RNA was isolated from the LV using TRIzol (Invitrogen, #15596018, ThermoFisher Scientific, Waltham, MA, United States) from hearts (apex) harvested at 28 days post-IR (5 samples/group; ND sham, 50 cGy simGCRsim-, and 100 cGy γ-IR). cDNA was synthesized using the cDNA synthesis kit (Applied Biosystems, #4368814, ThermoFisher Scientific, Waltham, MA, United States) according to the manufacturer’s instructions. Real-time quantitative PCR (RT-qPCR) was performed using PerfeCTa SYBR Green FastMix (Quantabio, #95074-012, Beverly, MA, United States) according to the manufacturer’s protocol. mRNA expression was normalized to GAPDH. Primer sequences are listed in Table 1a.

**TABLE 1a:** List of primers for genes involved in cardiac remodeling.

Gene	Primers (5′–3′)
***Tgfβ1***	F-CCTGCAAGACCATCGACATGGAG
	R-GGTCGCGGGTGCTGTTGTA
***Col1a1***	F-CTGGCAAGAAGGGAGATGA
	R-CACCATCCAAACCACTGAAA
***Col3a1***	F-GATGGAAACCCTGGATCAGA
	R- GCA​CCA​GGA​GAA​CCA​TTT​TC
***NCX***	F-TTTGCCTTCGTCCCACCTAC
	R-AACGGCAGTCACGGAATCTT
***Serca2a***	F-ACGCCTGCAACTCGGTCATA
	R-ATGTCCGGCTTGGCTTGTTT
***Tnfα***	F-CAAGTGGAGGAGCAGCTGGA
	R-CTGACGGTGTGGGTGAGGAG
***Mcp1***	F-TGCAGGTCCCTGTCATGCTT
	R-TCTTTGGGACACCTGCTGCT
***Mmp9***	F-CGCTCATGTACCCGCTGTAT
	R-CCGTGGGAGGTATAGTGGGA
***Gals3***	F-ACAGTCAGCCTTCCCCTTTG
	R-GTTAGCGCTGGTGAGGGTTA
***βMHC***	F-ACTGTCAACACTAAGAGGGTCA
	R-TTGGATGATTTGATCTTCCAGGG
***ANP***	F-GCTTCCAGGCCATATTGGAG
	R-GGGGGCATGACCTCATCTT
***BNP***	F-CTGGAAGTCCTAGCCAGTC
	R-TTTTCTCTTATCAGCTCCAGCA
***GAPDH***	F-GTGAAGGTCGGTGTGAACG
	R-TCGTTGATGGCAACAATCTC

***Vascular plaque burden.*** Aortas and branches (brachiocephalic, carotid, and subclavian arteries) were harvested at 365, 440, and 660 days post irradiation (Figure 1A). To assess vascular plaque burden, *en face* dissected vasculature was fixed in 10% formalin and kept overnight at 4°C. Subsequently, aortas were washed with PBS and again stored at 4°C overnight. In preparation for Oil Red O (ORO) staining, aortas were submerged in 100% propylene glycol for 2 min and then transferred to 0.5% ORO dye (Oil Red O Stain Kit, Abcam, Cat: ab150678) and incubated at 60°C for 50 min. Aortas were washed three times for 4 min with 85% propylene glycol, followed by a final wash with 1x PBS at 4°C overnight while gently agitated on a shaker. ORO-stained aortas were imaged using an 18 Megapixel camera.

***Modeling code and software.*** Data visualization, analysis, and statistics were performed using custom-written scripts in Python Version 3.7.6 (Python Software Foundation; < http://www.python.org >). Supporting packages/libraries included csv, glob, intertools, matplotlib, numpy, os, pandas, pylab, pynverse, researchpy, scipy, seaborn, and statsmodels. Dose-response curve fitting was performed using Benchmark Dose Software (BMDS) Version 3.1.2, developed by the U.S. Environmental Protection Agency (EPA) for risk analysis (https://www.epa.gov/bmds).

***Dose-response modeling.*** Models were fit for LVEF, LVFS, and DWS data that showed statistical significance with ANOVA and *post hoc* testing. The BMDS Exponential, Hill, Polynomial, Power, and Linear models were run for continuous endpoints. Best fit models were selected by—1) comparing model fits that showed the lowest AIC (Akaike Information Criterion); 2) displaying a goodness-of-fit below a value specified by BMDS; 3) exhibiting scaled residual values that did not exceed an absolute value of 2; and 4) visually evaluating the model curves which showed the most biologically relevant shape given the data resolution.

The Frequentist Exponential degree 4 (Freq. Exp. Deg. 4) model was selected as the best fit to represent the LVEF and LVFS data:

$$model\left(d\right)=a*\left(c-\left(c-1\right)*e^{-b*d}\right)$$ (1)

where *a* is the control response (intercept), *b* is the slope, *c* is the asymptote term, and *d* is the dose.

The linear model was selected as the best fit to represent the data for DWS:

$$model\left(d\right)=g+\beta_{1}*d$$ (2)

where *g* is the control (intercept), *β*_1_ is the polynomial coefficient, and *d* is the dose.

To produce dose-response models, LVEF and LVFS were dichotomized into categories of “normal” and “diseased” which represents altered cardiac function that may present with symptoms of developing heart failure. Cutoffs were assigned for both parameters as follows: LVEF of 40%–100% is “normal” while <40% is “diseased” and LVFS of 30%–60% is “normal” while <30% is “diseased.” Dose-response models were fit to the dichotomized data for each dataset that showed statistical significance with ANOVA and *post hoc* testing. Dichotomized dose-response models were analyzed but were not used in subsequent analyses (reasoning given in Results section “*Dose-response models with dichotomized data”*).

***RBE and RER calculations.*** Selected BMDS dose-response models were used to calculate the RBE and RER to compare the γ- and simGCRsim-IR functional cardiac responses. Only datasets that showed statistical significance between dose cohorts with hypothesis testing were modeled in BMDS and used for calculations. Lower LVFS and LVEF values (represented as a percent) indicate more adverse outcomes; the complement of the percentage was taken as the response value before the comparison ratios were calculated. This adjustment allowed the RBE and RER to be directly interpreted as surrogate markers of risk.

The RBE is the ratio between the radiation doses for a given biological response level. Given the response is the output of the original BMDS model (written as a function of dose that reports the response), the model inverse was used, which was configured as a function of response that reports the dose.

With the previous adjustments, RBE was calculated as follows:

$$RBE=\frac{D_{\gamma }}{D_{I}}$$ (3)

where D_γ_ is the dose for γ-IR and D_I_ is the dose of simGCRsim-IR at a given response. The RBE was reported as a function of the simGCRsim IR dose.

The RER calculates the ratio of the responses observed between the compared IR types for a given dose (Berrington et al., 2001). Note that the definition here is the inverse of that Shuryak et al., 2017. This was done to be consistent with the previous results (Brojakowska et al., 2023) and this difference will need to be accounted for when using this data to develop radiation quality models. Since the BMDS models are a function of dose and report the biological response, no adjustment was required prior to calculation. The RER was calculated as follows:

$$RER=\frac{R_{\gamma }}{R_{I}}$$ (4)

where R_γ_ is the biological response to γ IR and R_I_ is the biological response to simGCRsim-IR for a given dose.

***Statistics for echocardiography and PCR.*** The echocardiography parameters for the ND sham, WD sham, and both IR groups were compared using one-way ANOVA with the Tukey *post hoc* test, given that the data passed the assumptions required for normality (Shapiro-Wilk) and heteroscedasticity (Brown-Forsythe). The *p*-values were calculated using a one-way ANOVA and Tukey *post hoc* test for multiple comparisons. All analyses were performed using GraphPad Prism 8, version 8.4.3 (GraphPad Software, Inc, La Jolla, CA, United States). Data are expressed as mean ± standard error of the mean (SEM), except in box plots where the whiskers extend from the minimum to the maximum. Differences were considered statistically significant at *p* < 0.05.

***Statistics for modeling.*** Normal data distribution was determined with an array of data visualizations and tests for normality (Kolmogorov-Smirnov Test and Shapiro-Wilk Test), Skew, and Kurtosis. Homoscedasticity was confirmed with Levene’s Test. Subsequently, one-way ANOVA was run to determine statistical significance between doses for a given mouse strain, IR type, and time point. ANOVA hypothesis testing was followed up with the Tukey HSD *post hoc* test for datasets that showed significance with ANOVA to distinguish significance between the control and IR cohorts.

## Results

**FIGURE 2:**
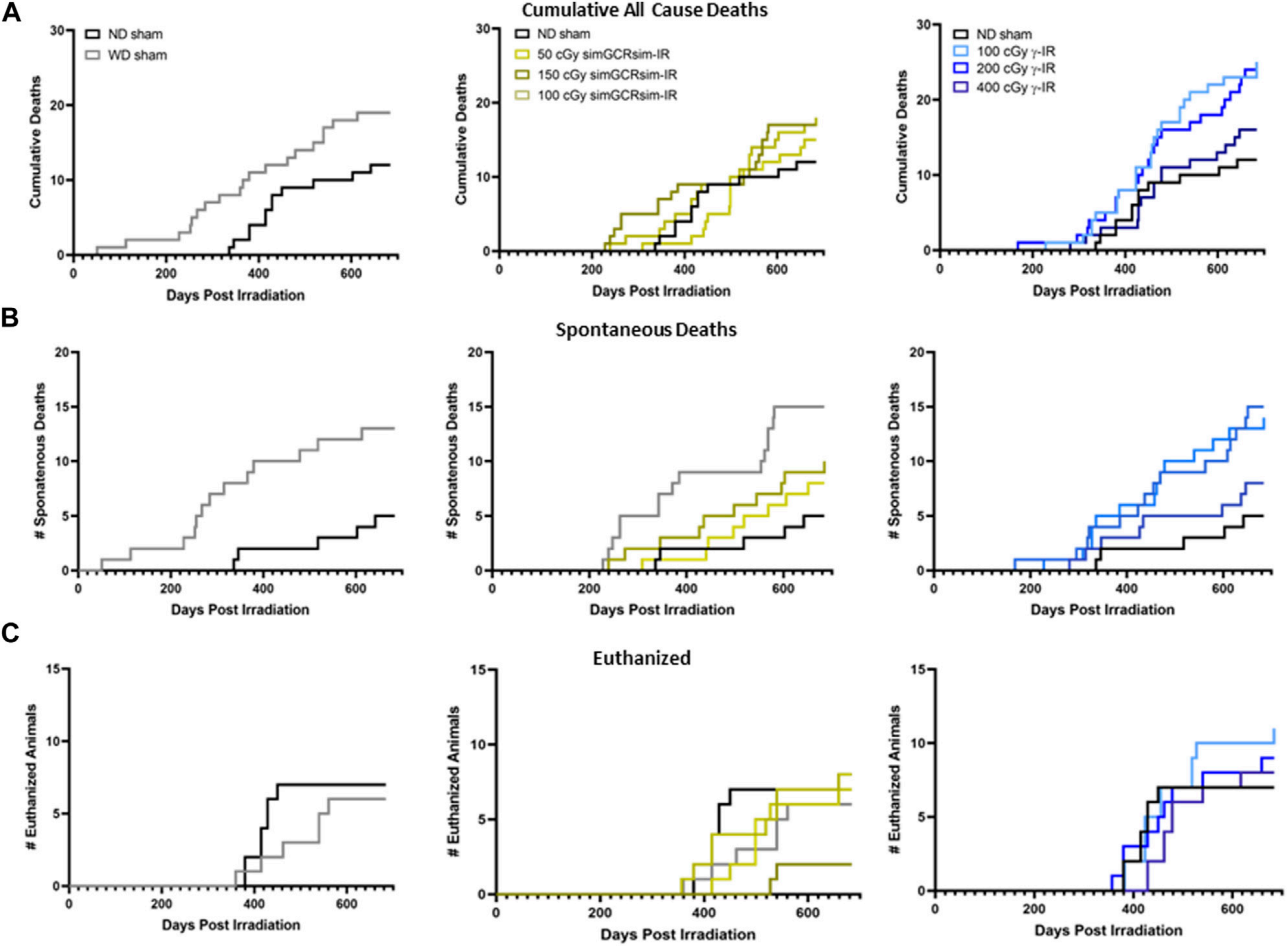
Mortality in ApoE Null male mice irradiated with gamma γ- and simGCRsim-IR. Graphs depicting a total number of **(A)** all-cause deaths, including **(B)** spontaneous deaths, defined as mice found dead in cages, and **(C)** animals euthanized at the designated collection timepoints due to adverse health effects.

***Effects of Irradiation on Physical Status and Mortality.*** ApoE null male mice were systematically assessed throughout the study for any effects of radiation on overall development and mortality (Figures 1A,B). There was no significant difference in heart rates between treatment groups during the acquisition of echocardiography data, suggesting that alterations in the heart rate were noncontributory to the obtained measurements (Figure 1C). Additionally, there was no significant difference in overall body weight or heart weight to body weight ratios in sham or irradiated mice across all time points (Figures 1D,E). Compared to ND-fed mice (ND sham), the total all-cause deaths (including spontaneous deaths and conditions requiring euthanasia) were higher in WD-fed non-irradiated mice (WD sham) and all irradiated mice (Figures 2A,B). Within simGCRsim-IR mice, mortality, particularly spontaneous death, was greater at the highest dose (150 cGy) compared to ND sham (Figure 2B, middle graph). Conversely, overall mortality was highest at the lowest and Intermediate doses of γ (Figure 2B, far right). Although necropsies did not reveal any definitive cause of death, all three doses of γ-IR mice (2-4 in each γ-IR group) showed marked multifocal atherosclerosis, myocardial fibrosis, hypertrophy, and to a lesser degree, increased evidence of potential toxic hepatic injury and inflammation (Supplementary Figure S2). Extensive multifocal atherosclerosis was also noted in simGCRsim-IR mice (2-4 in each simGCRsim-IR group). A very small number of mice (unequally spread among the groups of 1-2 in each group) also have an increased incidence of possible infectious disease processes as well as cancer (lymphoma, alveolar carcinoma, hepatocellular carcinoma, histiocytic sarcoma) (Supplementary Figure S2). Note all above necropsy findings were found predominately in γ- or simGCRsim-IR with only 2 necropsy findings of marked, multifocal extensive atherosclerosis in ND sham and one of each metastatic lymphoma and severe diffuse vacuolar degeneration of liver in WD sham groups each. Additionally, there was no evidence of noticeable pulmonary edema at time of sacrifice or in mice found prematurely dead and autopsied.

**FIGURE 3:**
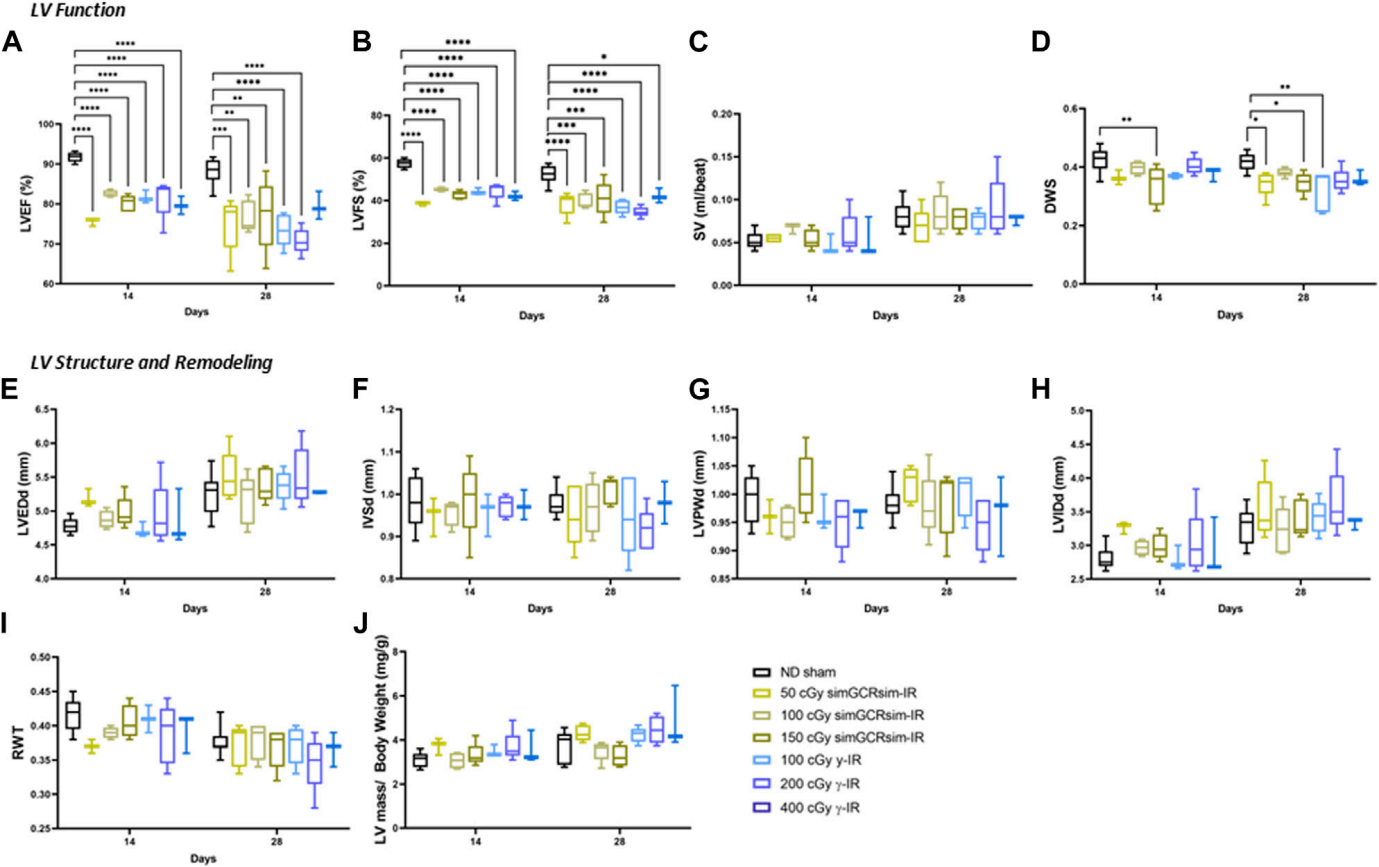
Longitudinal echocardiography results in ApoE-null male mice at 14 and 28 days after gamma (γ) and simGCRsim irradiation. Three-month-old male ApoE null mice were irradiated with simGCRsim-IR (50, 100, and 150 cGy) or gamma (γ)-IR (100, 200, and 400 cGy). Left Ventricular (LV) function is represented by **(A)** ejection fraction (EF), **(B)** fractional shortening (FS), **(C)** stroke volume (SV), and **(D)** diastolic wall strain (DWS). Parameters of LV dimensions and remodeling are represented by **(E)** LV end-diastolic diameter, **(F)** intraventricular septal thickness, **(G)** LV posterior wall thickness, **(H)** LV internal cavity diameter, **(I)** relative wall thickness, and **(J)** LV mass normalized to body weight. No-IR normal-diet (ND)-fed mice served as controls. For 14 days *N* = 3-9 animals (ND sham N = 9, 50 cGy simGCRsim and 100, 400 cGy γ *N* = 3, 100 cGy simGCRsim *N* = 4, 150 cGy simGCRsim and 200 cGy γ-IR *N* = 5) and 28 days N = 5 (except 400 cGy γ N = 3). *p*-values were calculated using one-way ANOVA. ****p < 0.0001, ***p < 0.001, **p < 0.01, *p < 0.05.

***Acute effects of irradiation on LV function.*** To assess the acute effects of simGCRsim- and γ-IR on LV function, echocardiography was performed at 14- and 28-days post single whole-body IR (Figures 3A–J). These early time points are considered acute “in-flight” time surrogates, as −1 month of mouse life equates to −2.9 years of human life, which is the time estimated for future Mars missions. Echocardiography provides a non-invasive modality to assess overall cardiac function and structural changes that may be physiological to maintain adequate cardiac output (CO) or could become pathological if it persists for a longer time (Gardin et al., 1995). Note, LV systolic function corresponds to the contractile capacity of the LV to maintain adequate CO, which is dependent on both stroke volume (SV) and heart rate. Systolic function is classically assessed by the LV ejection fraction (LVEF) (i.e., the change in blood volume within the LV between systole and diastole). LV fractional shortening (LVFS), an additional marker of LV contractility, describes the change in LV dimension between systole and diastole. Both LVEF and LVFS are affected by cardiac preload and afterload, and both can inform the overall systolic function of the heart. It is important to note that LVEF within the normal range cannot exclude the presence of diastolic dysfunction, which also has compensatory physiological consequences regarding the maintenance of CO.

As early as 14 days post-IR, global systolic function is significantly decreased in both IR groups across all doses (*p* < 0.001) (Figures 3A,B). Despite impaired systolic function, SV was not significantly different between irradiated and ND sham mice, suggesting that compensatory mechanisms may be present to preserve CO (Figure 3C). In 150 cGy simGCRsim-IR mice, the −13% decline in LVEF (80.2 ± 1 vs. ND sham 91.7 ± 0.4; *p* < 0.001) and −15% decline in LVFS (42.7 ± 0.9 vs. ND sham 57.4 ± 0.7; *p* < 0.001) was also associated with a decline in diastolic wall strain (DWS) (Figure 3D). DWS index reflects impaired diastolic wall thinning that leads to LV resistance to diastolic deformation based on the elastic theory (Ohtani et al., 2012), suggesting that the highest dose simGCRsim-IR mice may have increased LV myocardial stiffness, which could reflect impaired contractility and, thus, systolic and diastolic function. No other significant structural alterations were observed at this early time point (Figures 3E–J).

At 28 days post-IR, ApoE null male mice continued to have persistently decreased global systolic function in all IR groups (*p* < 0.05) (Figures 3A,B). Similar to 14 days post-IR, there was no significant difference in SV nor structural parameters such as LV end-diastolic dimension (LVEDd), intraventricular septal (IVSd), or posterior wall thickness (LVPWd) (Figures 3C, E–J). Interestingly, DWS was significantly decreased in both 50 and 150 cGy simGCRsim-IR mice and 100 cGy γ-IR mice (*p* < 0.05) (Figure 3D), suggesting that by 28 days post IR more pathologic changes may have accumulated in response to IR exposure to alter LV compliance. It is also important to consider the significant inter-animal variability observed.

**FIGURE 4:**
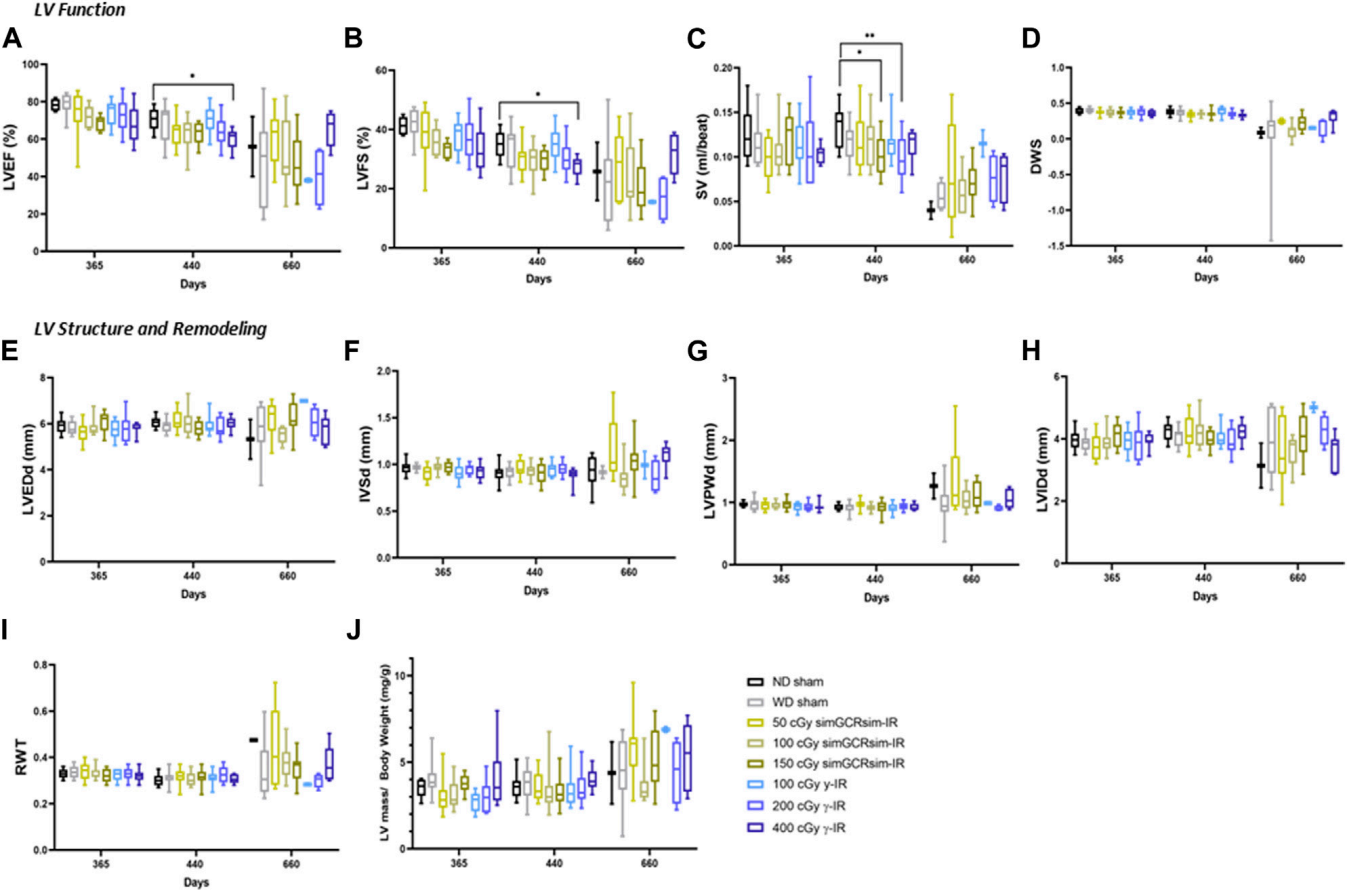
Longitudinal echocardiography results in C57BL/6J male mice at 365, 440, 660 days after γ- and simGCRsim-IR. Left Ventricular (LV) function is represented by **(A)** ejection fraction (EF), **(B)** fractional shortening (FS), **(C)** stroke volume (SV), and **(D)** diastolic wall strain (DWS). Parameters of LV dimensions and remodeling are represented by **(E)** LV end-diastolic diameter, **(F)** intraventricular septal thickness, **(G)** LV posterior wall thickness, **(H)** LV internal cavity diameter, **(I)** relative wall thickness, and **(J)** LV mass normalized to body weight. *N* = 7–10 animals for 365 & 440 days and N = 2–10 (ND sham and 100 cGy γ-IR *N* = 2, remaining N ≥ 4); *p*-values were calculated using one-way ANOVA. **p < 0.01, *p < 0.05.

***Degenerative effects of irradiation on LV function.*** To assess the long-term (degenerative) effects of simGCRsim- and γ-IR on LV function, we performed echocardiography 365-, 440-, and 660-days post-IR (Figures 4A–J). At 365 days post-IR, there was no significant difference in LV systolic function or structure detected by echocardiography. By 440 days post-IR, there was an overall subtle decline in global systolic function, defined by LVEF and LVFS, in both controls (ND and WD sham) and all irradiated groups, which may reflect an age-related decline in overall LV function. Among all groups, global LV systolic function was significantly reduced at the highest dose of γ-IR (400 cGy) compared to ND sham, with a ∼10% decrease in LVEF (60% ± 2 vs. ND sham 70.6% ± 1.7; *p* < 0.05) and ∼8% decline in LVFS (27.5% ± 1.2 v. ND sham 35% ± 1.3, *p* < 0.04) (Figures 4A,B). This decline in systolic function was not associated with any detectable structural alterations (Figures 4E–J). Although the reduction in LVEF and LVFS were not statistically significant, both 150 cGy simGCRsim and 200 cGy γ-IR mice had a reduced SV compared to ND sham, suggesting that LV function may be impaired through alternative mechanisms (i.e., changes in diastolic function, afterload, and preload) not assessed by standard M-mode echocardiography (Figures 4A–C). There was no significant difference in the overall LV structure and function at 660 days, although the limited survival of ND sham mice (N = 2) at this time limits the statistical power, as well as may introduce a potential survival bias at this timepoint. Additionally, considerable inter-animal variability was noted particularly at this last time point, which was potentially magnified by age-related changes associated with animals approaching the end of life.

**FIGURE 5:**
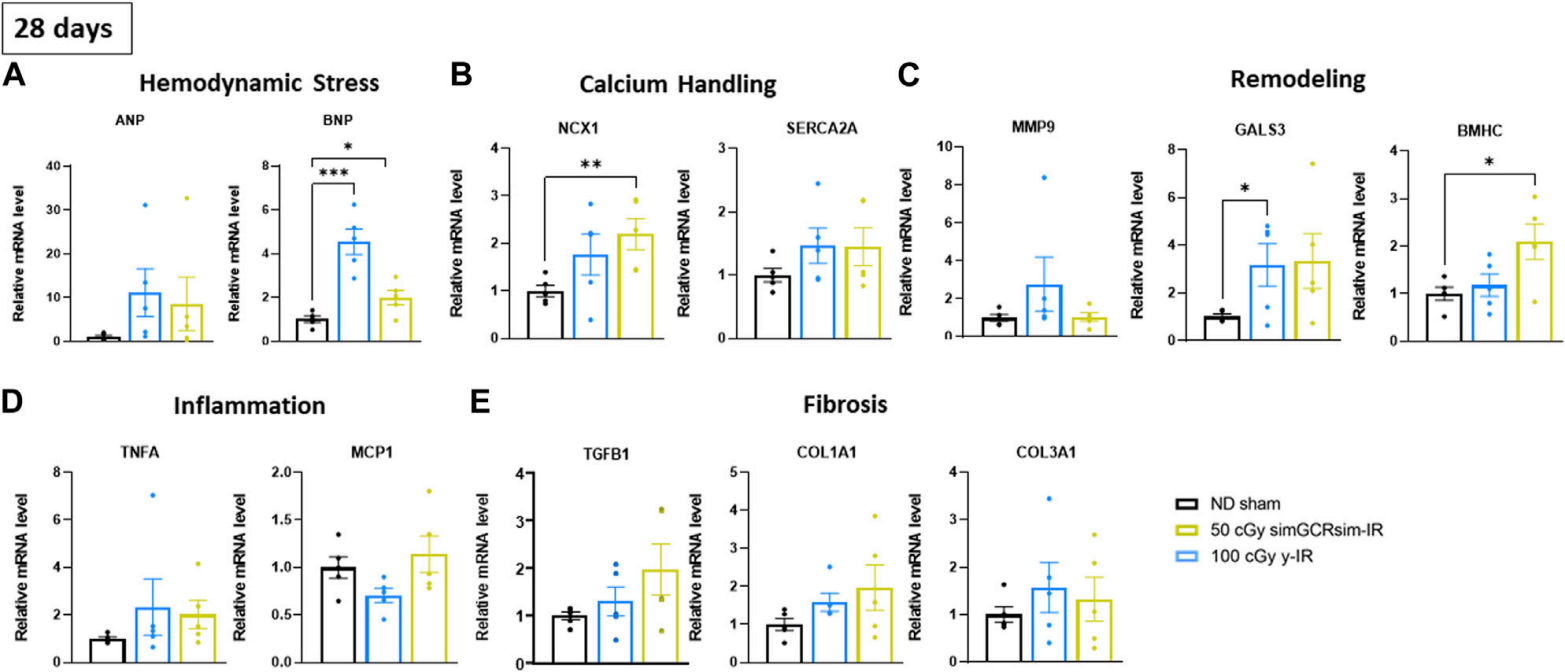
Gene expression in left ventricular heart tissue (apex) was assessed 28 days after simGCRsim- and γ-IR. Relative mRNA expression of **(A)** hemodynamic stress (*ANP, BNP*), **(B)** calcium handling protein (*NCX*, *SERCA2a*), **(C)** cardiac remodeling (*Mmp9, Gals3, βMHC*), **(D)** inflammation (*Tnfα, Mcp1*), and **(E)** cardiac fibrosis (*Tgfβ1, Col1a1, Col3a1*) markers within the left ventricle (LV) of mice irradiated with 50 cGy simGCRsim or 100 cGy γ at 28 days post-irradiation. *Tgfβ1* indicates transforming growth factor beta 1; *Col1a1*, type I fibrillar collagen; *Col3a1*, type III fibrillar collagen; *Ncx*, cardiac sodium-calcium exchanger; *SERCA2a*, Sarcoplasmic Reticulum Ca^2+^ ATPase; *Tnfα*, tumor necrosis factor alpha; *Mcp-1*, monocyte chemoattractant protein-1; *Mmp-9*, matrix metalloproteinase-9; *Gals3*, galectin 3; *βMHC*, cardiac beta myosin heavy chain; *ANP*, atrial natriuretic peptide; *BNP*, brain natriuretic peptide. Each dot represents an individual mouse. Expression was normalized to *GAPDH*. Data reported as mean ± SEM. N = 5 animals/treatment group; *p*-values were calculated using a one-way ANOVA and Tukey *post hoc* test. ***p < 0.001, **p < 0.01, *p < 0.05.

***Changes in the expression of cardiac markers of hypertrophy, fibrosis, and inflammation.*** Considering that the most significant changes in LV function were observed within the acute “in-flight” stage post-IR, we assessed the expression of various markers of cardiac remodeling and inflammation in ApoE-null ND sham, 50 cGy simGCRsim- and 100 cGy γ-IR mice 28 days post-IR (Figures 5A–E). Given that no difference in response was noted between different doses for each given radiation type, we examined the hearts isolated from ApoE-null mice at the lowest dose of simGCRsim- and γ-IR. We assessed the expression of hemodynamic markers atrial natriuretic peptide (*ANP*) and brain natriuretic peptide (*BNP*), whose elevation is a hallmark of physiological compensatory response to heart failure (Reginauld et al., 2019). Although not statistically significant, transcript levels of *ANP* were elevated in both IR groups (Figure 5A). However, transcript levels of *BNP* were significantly elevated in both IR groups compared to ND sham, with a 4.2-fold higher levels in simGCRsim- and 2-fold increase in γ-IR (*p* ≤ 0.001 and *p* ≤ 0.05, respectively), suggesting systolic dysfunction observed at 28 days post IR in both IR groups resulted in significant hemodynamic stress.

Calcium handling is an essential mediator of cardiac contractility and its dysfunction can contribute to mechanical and electric cardiac dysfunction (Berridge et al., 2003). Sarcoplasmic Reticulum Ca^2+−^ATPases (*SERCA2a*) and Na^+^/Ca^2+^ exchangers (*NCX*) provide a critical regulatory domain for maintaining intracellular Ca^2+^ homeostasis following Ca^2+^-triggered troponin-C contraction, determining the rate, intensity and duration of myocyte contraction (Yue et al., 1986). Transcript levels of *NCX* were significantly elevated in γ-IR mice compared to ND sham mice (Figure 5B) (*p* ≤ 0.01). Additionally, although not statistically significant, transcript levels of *SERCA2a* were elevated in both IR groups compared to ND sham mice, suggesting that impaired intracellular Ca^2+^ handling may contribute to observed systolic dysfunction in irradiated mice.

Furthermore, we assessed the expression of genes involved in various cardiac remodeling processes. There was no significant difference in transcript levels of matrix metallopeptidase 9 (*Mmp9*), involved in remodeling the extracellular matrix and reported to be elevated post-myocardial infarct cardiac remodeling (Ertl and Frantz, 2005), though the overall levels were highest in γ-IR mice (Figure 5C). Additionally, there was a significant increase in the expression of galectin-3 (*Gals3*), a marker of cardiac fibrosis and inflammation in various CVDs (Dong et al., 2018), in simGCRsim-IR mice (*p* < 0.05). In contrast, γ-IR ApoE null mice had elevated levels of *Gals3 (*p = ns*)* and a 2-fold increase (*p* ≤ 0.05*)* in the expression of beta-myosin heavy chain (*ßMHC*), a marker of cardiac hypertrophy that is more pronounced in pressure overload (Gupta, 2007), suggesting γ-IR may induce different from simGCRsim pathways involved in cardiac remodeling (Figure 5C). There was no significant difference in the expression of inflammatory markers - tumor necrosis alpha (*Tnfα*) or monocyte chemoattractant protein (*Mcp1*) (Figure 5D). Additionally, there was no significant difference in the expression of cardiac fibrosis markers, such as transforming growth factor-β1 (*Tgfβ1*), type I (*Col1a1*), and type III fibrillar collagen (*Col3a1*), in any of the IR groups (Figure 5E).

**FIGURE 6:**
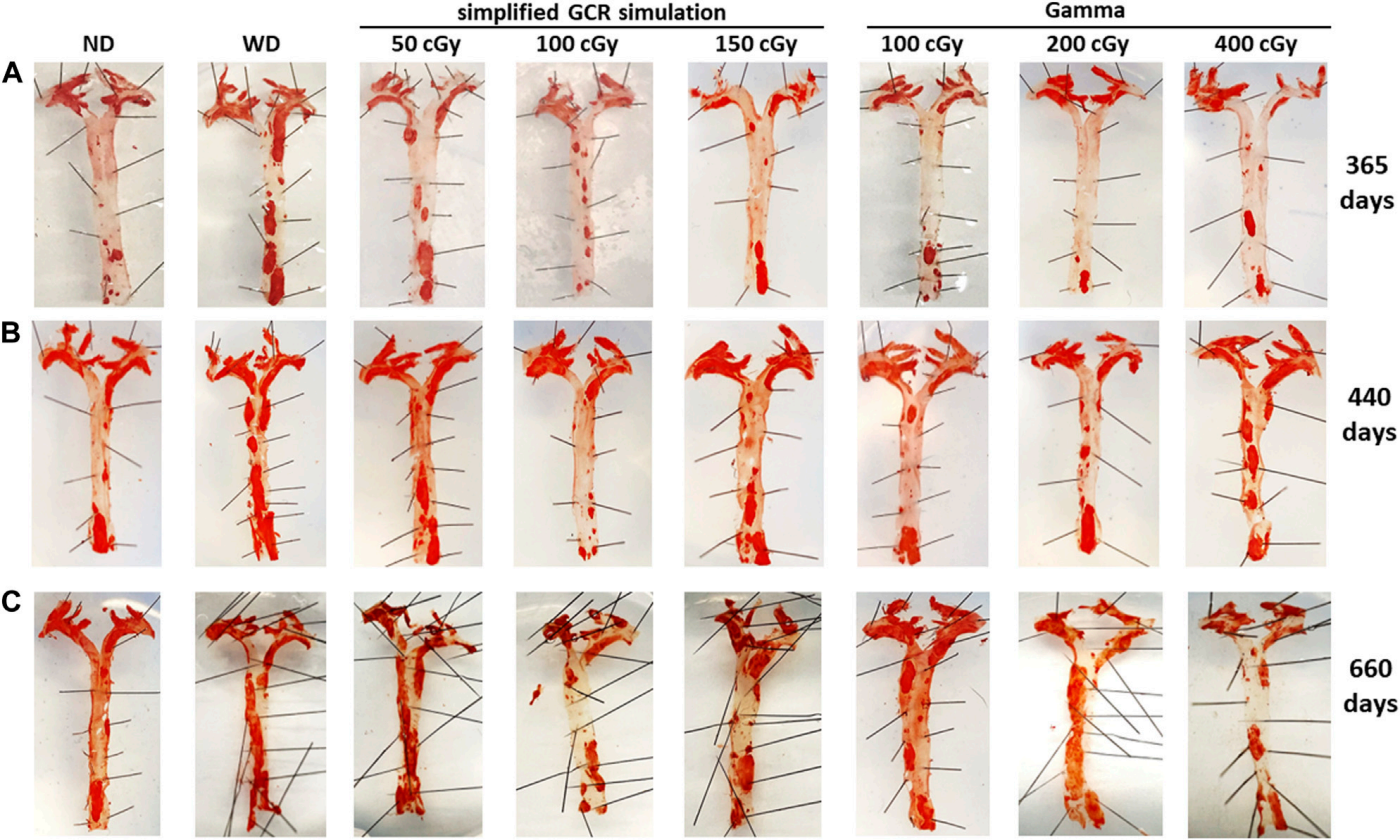
Vascular plaque burden in *en face* aortas 365, 440, 660 days post irradiation with gamma and simGCRsim-IR. Aortas and branching arteries (brachiocephalic, carotids, subclavian) were harvested from mice and stained with Oil Red O to assess for vascular plaque at **(A)** 365, **(B)** 440, and **(C)** 660 days post irradiation with varying doses of simGCRsim (50, 100, 150 cGy) and gamma (100, 200, 400 cGy). Nonirradiated normal diet (ND) and western diet (WD) fed mice were negative and positive control, respectively.

***Vascular plaque burden in aortas and aortic branches.*** To assess the effects of irradiation on vascular plaque burden, aortas and aortic branches (brachiocephalic, carotids, subclavian arteries) were harvested and stained with Oil Red O (Figure 6). At 365 days post IR, noticeable plaque is localized at the ascending aorta and its branches across all treatment conditions (Figure 6A). However, in WD sham and low dose (50 cGy) simGCRsim-IR mice, there is more observable saturation of plaque along the descending aorta. As expected with aging, there is progressive plaque accumulation, with saturation at the aortic arch and bifurcating arteries, at 440 days post IR in both ND sham and all treatment conditions; however, aortas of WD sham mice, 50 cGy simGCR-sim, and 400 cGy γ-IR ApoE null mice appear more saturated along the descending segments in comparison to remaining conditions (Figure 6B). By 660 days post IR, considerable plaque is noted along all segments of the aorta in all treatment conditions; however, plaque burden appears higher in WD sham mice, 50 cGy simGCRsim-IR mice, and at both low doses of γ (100, 200 cGy) (Figure 6C). A progressive increase in plaque at both low-dose γ-IR aortas at 660 days suggests there is a point where the effects of IR and aging intersect to present a similar plaque phenotype to WD sham mice.

**FIGURE 7:**
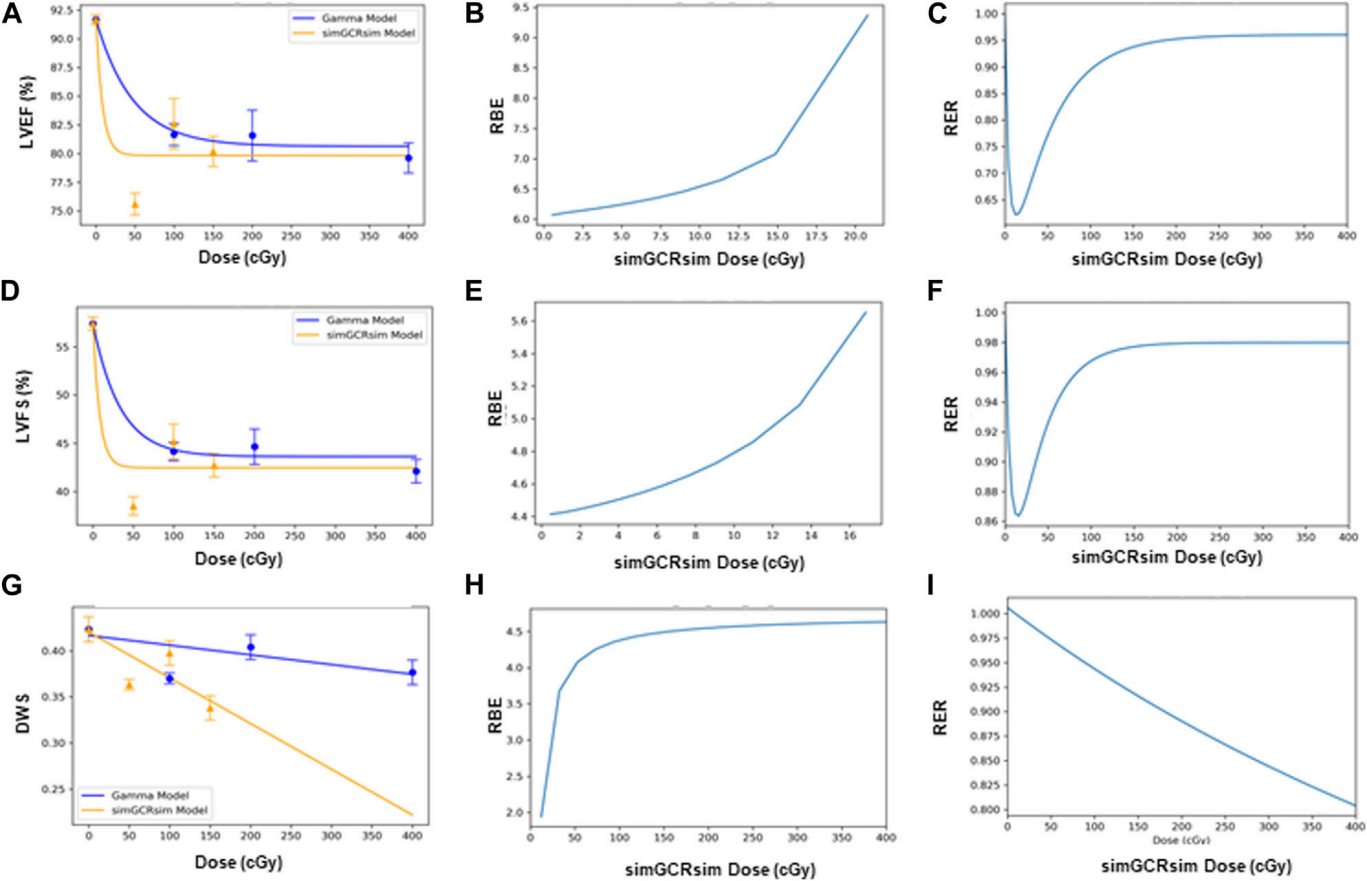
Modeling and comparative effect measures of ApoE-null male mice 14 days after exposure to gamma and simGCRsim irradiation. Comparison of **(A)** LVEF, **(D)** LVFS, and **(G)** DWS mean dose-response data (SEM error bars) superimposed on BMDS dose-response models for simGCRsim (orange triangles) and gamma (blue circles) irradiation. To the right are the corresponding RBE calculation within the viable range **(B)**, **(E)**, and **(H)**, and the corresponding RER calculation **(C)**, **(F)**, and **(I)** for LVEF, LVFS, and DWS respectively.

***BMDS dose-response modeling.*** The LVEF and LVFS show an initial drop in systolic function with dose, which then flattens out. This dose-response shape was best fit by the Freq. Exp. Deg. 4 (example shown in Figures 7A,D). These models reported Benchmark Doses (BMD), a dose level that indicates where the risk increases above background by some predefined level, that are very low or near zero. BMD is an estimate of the threshold dose for the radiation response. However, because the BMD values were so low, if a threshold does exist, it would be lower than the lowest radiation dose that was measured (50 cGy simGCRsim- or 100 cGy γ-IR). The DWS dose-response was best fitted by a linear model, which, by virtue, did not exhibit a threshold (example shown in Figure 7G).

***Dose-response models with dichotomized data.*** When the data was dichotomized (normal: LVFS 30%–60%, LVEF 50%–100%; diseased: LVFS <30%, LVEF <50%) and modeled in BMDS, the results demonstrated a rather large uncertainty (expressed by large error bars which represent 95% CI on proportions observed) due to the large individual variation and small sample sizes. For LVFS, the large uncertainty was also influenced by the relatively large percentage of “individuals” who fell within the “diseased” category. This large uncertainty rendered the dichotomized data unsuitable for dose-response modeling. For LVEF, for most of the time points, very few, and sometimes not even a single “individual,” fell within the “diseased” range. As noted previously, dichotomization of LVEF is ineffective in recapitulating the full disease spectrum that may present in the setting of heart failure, including systolic heart failure, which is classically defined as a reduced LVEF *versus* diastolic heart failure with a preserved LVEF (Vedin et al., 2017; Simmonds et al., 2020). This lack of a strong response also did not lead the LVEF data to dichotomized dose-response modeling (data not shown).

**FIGURE 8:**
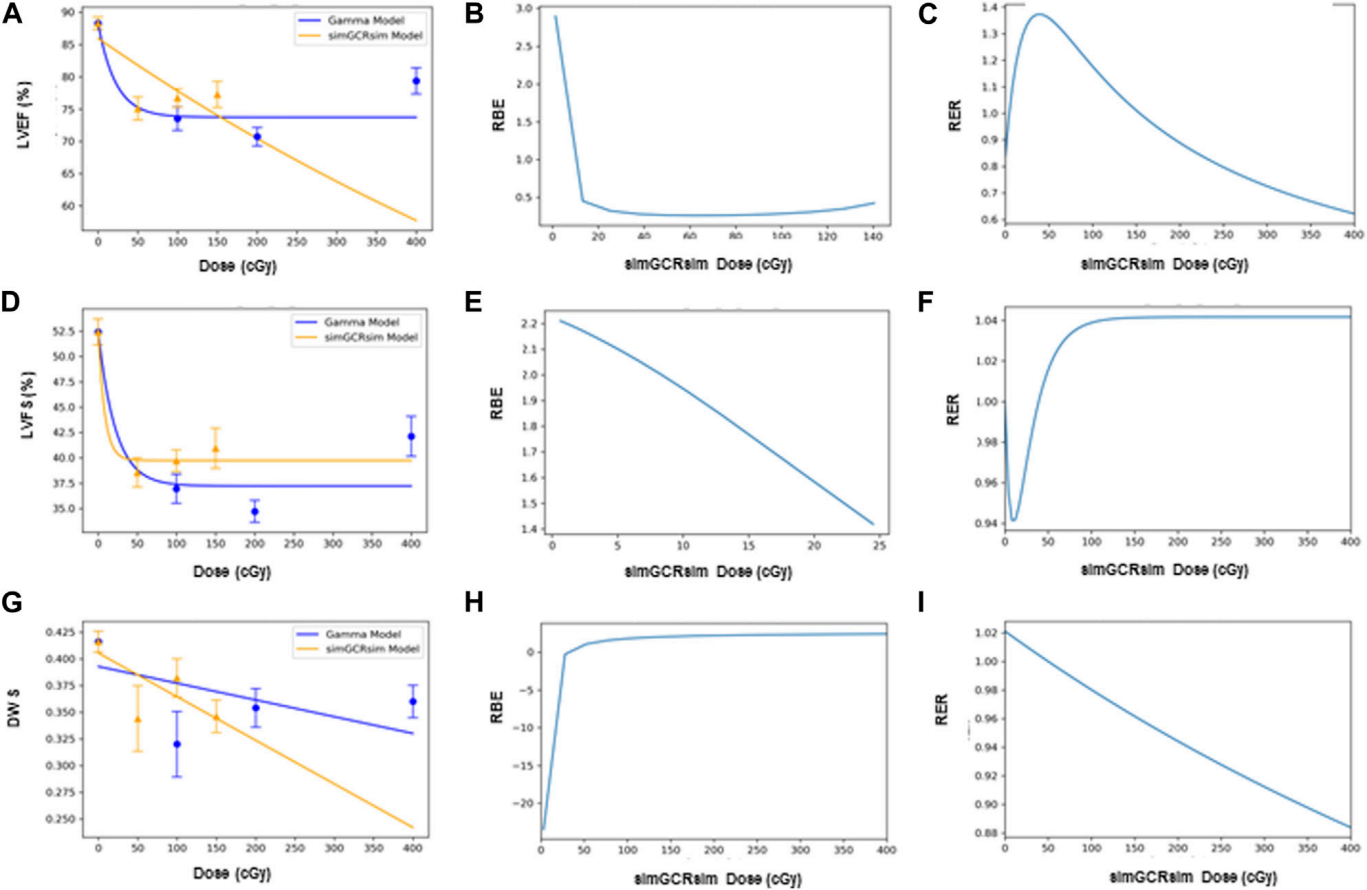
Modeling and comparative effect measures of ApoE-null male mice 28 days after exposure to gamma and simGCRsim irradiation. Comparison of **(A)** LVEF, **(D)** LVFS, and **(G)** DWS mean dose-response data (SEM error bars) superimposed on BMDS dose-response models for simGCRsim (orange triangles) and gamma (blue circles) irradiation. To the right are the corresponding RBE calculation within the viable range **(B)**, **(E)**, and **(H)**, and the corresponding RER calculation **(C)**, **(F)**, and **(I)** for LVEF, LVFS, and DWS respectively.

***RBE and RER calculations.*** The Relative Biological Effectiveness (RBE) is the ratio between the γ- and simGCRsim-IR doses, resulting in the same biological response. RBE is undefined over much of the dose range. More specifically, the interval an RBE could be calculated for the ranges of 16.84 cGy–50.77 cGy for LVFS and 20.79 cGy–140.54 cGy for LVEF (Table 1). Note, 140.45 cGy is the highest range that the RBE could be calculated for within the measured doses for the simGCRsim (Table 1). For LVFS, the Freq. Exp. Deg. 4 model gave a more linear fit to the day 440 of γ-IR data, which allowed the RBE to be calculated for the entire simGCRsim dose range. For DWS, the linear dose-response model best fits the data, which allowed the RBE to be defined along the entire range of simGCRsim IR doses measured (Table 1; Figures 7H, 8H).

**TABLE 1:** RBE values at the minimum and maximum simGCRsim dose range where the calculation was defined.

Response	Days after IR	RBE	Min SimGCRsim dose (cGy)	RBE	Max SimGCRsim dose (cGy)
**LVEF (%)**	14	6.070128	0.557324446	9.369474	20.78632113
	28	2.895274	1.390505729	0.421783	140.5444577
	440	9.606402	13.30313874	0.969099	8068.154144
**LVFS (%)**	14	4.413665	0.475966053	5.652795	16.83716906
	28	2.210004	0.622886578	1.418587	24.51164339
	440	20.6816	3.109901666	4.913137	50.77487307
**DWS**	14	1.94237	12.19310591	4.675021	848.4897299
	28	−23.4609	3.071050308	2.523941	992.7955142

**FIGURE 9:**
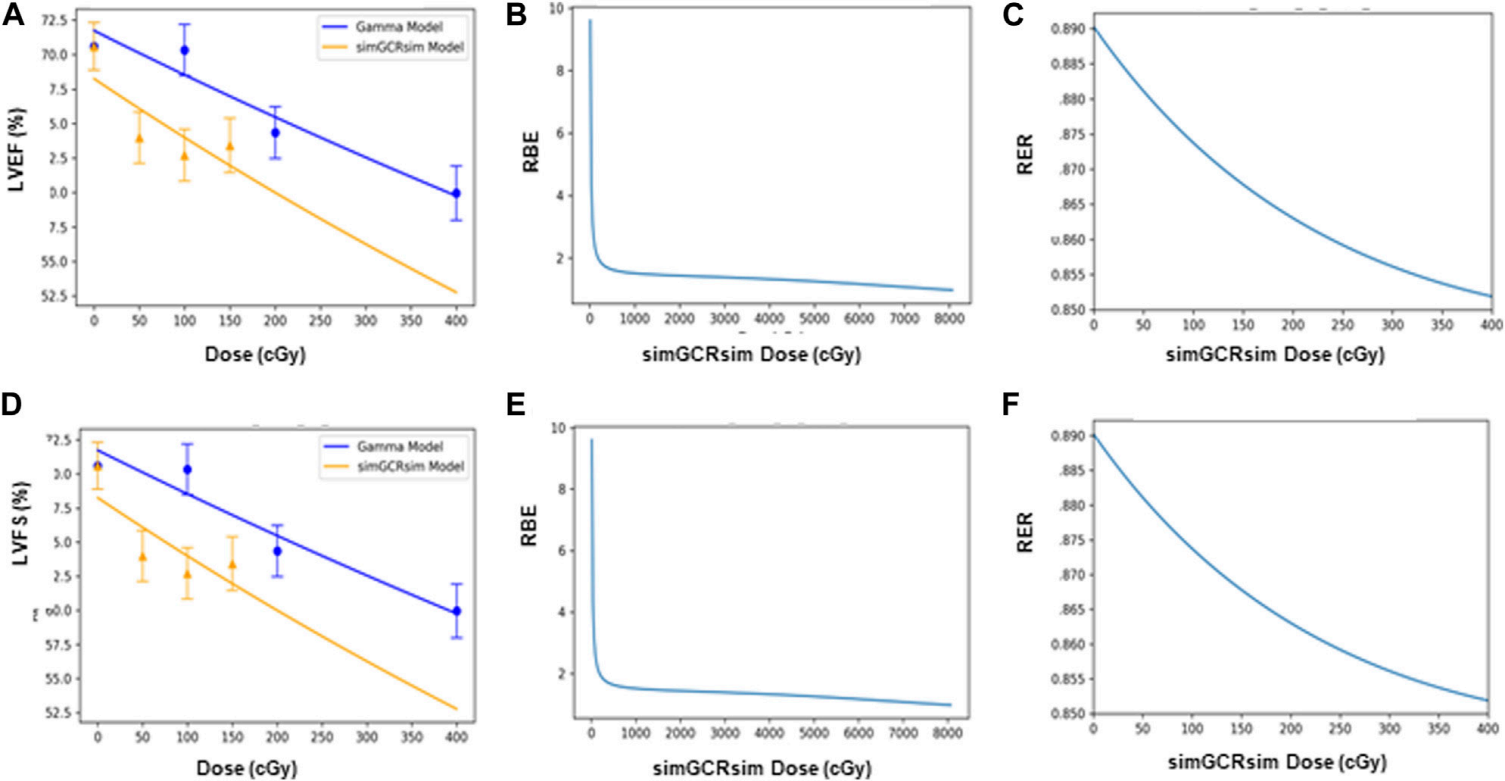
Modeling and comparative effect measures of ApoE-null male mice 440 days after exposure to gamma and simGCRsim irradiation. Comparison of **(A)** LVEF and **(D)** LVFS mean dose-response data (SEM error bars) superimposed on BMDS dose-response models for simGCRsim (orange triangles) and gamma (blue circles) irradiation. To the right are the corresponding RBE calculation within the viable range **(B)**, **(E)**, and the corresponding RER calculation **(C)**, **(F)** for LVEF and LVFS, respectively.

The Radiation Effects Ratio (RER) is the measured ratio between the biological effects observed with γ- and simGCRsim-IR at the same dose level. This allows the RER to be calculated along the entire dose range measured, regardless of the model shape and biological response level (compare the difference between Figures 7C,F,I; Figures 8C,F,I or Figures 9C,F and the corresponding dose-response models shown in Figures 7A,D,G; Figures 8A,D,J; Figures 9A,D). RER values were close to one when calculated at an irradiation dose of zero (Table 2), as expected, because both irradiation models were estimated at the same beginning control dose point. Beyond the control dose, the RER values vary minimally; RER values vary no more than 0.09 for LVFS, 0.74 for LVEF, and 0.20 for DWS across the dose points measured (Table 2).

**TABLE 2:** RER values at the measured gamma and simGCRsim dose levels.

		simGCRsim-IR dose (cGy)					
Response	Day after IR	0	50	100	150	200	400
**LVEF (%)**	14	0.999962	0.925997	0.967499	0.97708	0.979262	0.979903
	28	0.836828	1.358758	1.177047	1.011224	0.888739	0.621614
	440	0.890278	0.881116	0.873752	0.867817	0.863034	0.851873
**LVFS (%)**	28	1.000023	1.015582	1.038841	1.041347	1.041611	1.041642
	440	0.986225	0.937534	0.945606	0.960312	0.975461	1.031726
**DWS**	14	1.006124	0.973604	0.943644	0.915952	0.89028	0.803934
	28	1.021097	0.999954	0.980169	0.961616	0.944182	0.883836

## Discussion

Over the last few decades, there has been increasing evidence associating radiation exposure with increased CVD risk, ranging from myocardial to valvular pathologies, especially in correlation with patients receiving mediastinal radiation therapy (Heidenreich et al., 2003; Hoon et al., 2007; Weintraub et al., 2010; Barcellos-Hoff and Mao, 2016; Nielsen et al., 2017; Wang et al., 2020; Little et al., 2023). Astronauts have an increased risk of exposure to ionizing radiation, especially those with planned deep-space missions to the Moon and Mars. However, little is known about the acute and long-term degenerative CVD risks following exposure to space-type radiation, especially HZE radiation with high LET. While murine studies of various HZE exposures (e.g., ^1^H, ^56^Fe, ^16^O) show that IR results in impaired systolic function (Yan et al., 2014; Nelson, 2016; Se et al., 2019), limited studies have examined the effect of simulated space-type radiation, i.e., simGCRsim on LV function. Our team recently showed that wild-type C57BL/6J male mice irradiated with either simGCRsim- or γ-IR exhibit impaired LV systolic function as early as 14 days post-IR, which remains suppressed at 365 days post IR, but appears to potentially phenotypically change (i.e., a shift from systolic to diastolic dysfunction) by 660 days post simGCRsim-IR (Brojakowska et al., 2023). However, while C56BL/6J mice can potentially develop diet-induced atherosclerosis (Paigen et al., 1987), this model is not the best to recapitulate human conditions, such as a history of underlying lipid or metabolic disorders (both diet-induced and genetic) that need to be considered as confounding risk factors in humans (Bozkurt et al., 2016).

In this study, we irradiated 3 month old age-matched ApoE null male mice, which can develop both spontaneous and diet-induced atherosclerosis (Nakashima et al., 1994; Lo Sasso et al., 2016), to assess the acute and long-term effects of simGCRsim- (50, 100, 150 cGy) and γ-IR (100, 200, 400 cGy) on LV function and structure. This study aimed to (Li et al., 2009) determine the effects of γ- and simGCRsim-IR on LV function in atherosclerosis-prone mice; (National Library of Medicine, 2002); identify the disease spectrum and latency following single, whole-body IR exposure; (Reinders et al., 1999); determine whether dose thresholds for CVD endpoints exist; and (Heidenreich et al., 2003) compared the effects of γ- and simGCRsim-IR by calculating RBE and RER.

As stated earlier, the acute effects of space-type and terrestrial (γ) exposure on LV function were assessed at 14- and 28-days post IR, which are roughly “in-flight” time points as approximately 1 month of mouse life is equivalent to 2.9 years of human life, which is the estimated time for future missions to Mars. By 14 days post-IR, there was a significant decline in LV systolic function, detected by reduced LVEF and LVFS, within both IR groups across all doses. At this time, no significant structural alterations were noted except in 150 cGy simGCRsim-IR mice, which had reduced diastolic wall strain (DWS). The reduction in LV systolic function persisted at 28 days post-IR in all IR groups; however, the reduction in DWS also became prominent in both simGCRsim- (50 and 150 cGy) and γ-IR (100 cGy) mice, suggesting that simGCRsim-may induce earlier effects on LV wall stiffness than γ-IR at lower doses as well as higher doses. DWS correlates with both LV systolic function and diastolic mechanics but does not represent LV diastolic wall strain (Selvaraj et al., 2014). However, decreased DWS in patients with heart failure with a preserved ejection fraction (HFpEF) appears to be associated with poorer outcomes, given the combined association of abnormal systolic and diastolic cardiac mechanics (Attwood et al., 2002; Bruch et al., 2003). Additionally, adult survivors of childhood leukemia (males aged 22.2 ± 5.5 years) also have significantly lower DWS, despite a preserved LVEF, suggesting increased LV myocardial stiffness associated with myocardial fibrosis and impaired diastolic function (Li et al., 2017). While there was an acute reduction in systolic function early in both IR groups, we cannot exclude the possibility of combined diastolic dysfunction, which would require further targeted investigation for longitudinal evaluation of diastolic (dys)function.

Considering these pronounced acute effects, we evaluated the expression of various markers of hemodynamic stress and cardiac remodeling from LVs harvested at 28 days post-IR in mice irradiated with the lowest doses of simGCRsim- and γ-IR. At 28 days, there was a significant increase in the expression of *Bnp* in both IR groups, suggesting that the mice experienced hemodynamic stress. Elevated BNP levels occur in response to increased tension within the LV myocardium either because of increased volume or pressure to promote natriuresis. Clinically, the extent of BNP elevation often corresponds with the symptomatology of heart failure exacerbation, as cardiogenic volume or pressure overload contributes to increased pulmonary venous pressure and pulmonary capillary pressure, which shifts fluid into interstitial spaces of alveoli, ultimately presenting as shortness of breath, orthopnea, and paroxysmal nocturnal dyspnea in patients. Therefore, BNP is a key diagnostic and prognostic marker of cardiac hemodynamic stress and possible heart failure (HF) in development (Gaggin and Januzzi, 2013). While we did not observe any noticeable pulmonary edema on gross observation of lungs harvested at each time point or at points where a necropsy was performed, further studies from our lab are focused on the comprehensive assessment of effects of IR on pulmonary physiology and may give us more insight into corresponding physiology. However, in combination with the observed systolic dysfunction (reduced LVEF and LVFS), this suggests that simGCRsim- and γ-IR mice may develop significant and acute decreases in systolic heart function within 28 days post-IR.

Systolic dysfunction encompasses impaired LV contractility, which is governed by myocardial calcium homeostasis. Both IR groups showed an increase in the expression of *Ncx1*, though it is significantly increased in γ-IR mice. Cardiac sodium-calcium exchanger (NCX) is an essential regulator of intracellular ion homeostasis, and studies have suggested that NCX activity is increased in HF and may contribute to impaired contractility by depleting the Ca^2+^ content in the sarcoplasmic reticulum (Studer et al., 1994; Flesch et al., 1996; Armoundas et al., 2007). Sag et al. studied cardiac myocytes irradiated with X-rays (4, 20 Gy) and showed that irradiation acutely disturbs Ca^2+^ homeostasis due to increased formation of oxidative stress, which activates calcium–calmodulin (CaM)-dependent protein kinase II (CaMKII), which then triggers sarcoplasmic reticulum Ca^2+^ release via modification of ryanodine receptor 2 (RyR2) (Sag et al., 2013). Therefore, alterations in cardiac calcium handling may be an IR-specific mechanism for the heart failure in development, and a therapeutic route to consider utilizing to mitigate disease pathogenesis or manage clinical symptoms.

No significant differences were observed in the selection of inflammatory or fibrotic markers. However, *Gals3* and *βMHC* expression was increased in simGCRsim- and γ-IR mice, respectively, again highlighting potential IR-specific induced cardiac remodeling. Galectin-3 is a beta-galactoside binding protein involved in various cellular processes and has recently been identified as a prognostic biomarker for HF, given its role in the pathophysiology of HF (Gehlken et al., 2018). Prolonged galectin-3 expression is associated with increased activation of fibroblasts into matrix-producing fibroblasts, contributing to the development of myocardial fibrosis (de Boer et al., 2009). On the other hand, beta-myosin heavy chain (*βMHC*) is involved in cardiac hypertrophy (Kamisago et al., 2006; Homburger et al., 2016; Landim-Vieira et al., 2022). These studies highlight that IR-specific mechanisms may result in impaired LV function development but require further investigation to determine whether existing therapeutics (i.e., antiarrhythmics, diuretics, etc.) may help mitigate the development of acute cardiac dysfunction.

In comparison, there was no significant difference in LV function or structure in simGCRsim- or γ-IR mice at 365- or 660-days post-IR. However, 440 days post-IR appears to be an intermediate time point at which alterations in cardiac function are observed. These findings do not exclude the possibility of variability in the aging of individual mice in addition to the survivor bias at this late for mice (approaching end of lifespan) timepoint. While global LV systolic function was significantly reduced only in 400 cGy γ-IR mice, as noted by reduced LVEF and LVFS, both 150 cGy simGCRsim and 200 cGy simGCRsim-IR mice have a significantly decreased SV, suggesting possible impairment in LV contractility or increased afterload (i.e., resistance needed for the LV to overcome for blood to enter systemic circulation). While this study indicates early and late changes in systolic and possibly diastolic function following simGCRsim- or γ-IR, further studies by our team are investigating the effect of IR on vasculature and valves (histology, plaque burden and vulnerability, media/intima thickness, endothelial function, etc.), which could provide additional CVD endpoints and help delineate whether observed changes in SV may be related to changes in factors related to preload *versus* afterload. Thus far, qualitative assessment of vascular plaque burden in aortas and bifurcating arteries harvested from animals at 365, 440, and 660 days post IR suggests IR exposure accelerates plaque development in comparison to ND sham mice across all time points and appears more considerable in WD sham and 50 cGy simGCRsim-IR mice. Plaque burden progresses in γ-IR mice across time and surpasses that of ND sham mice by 660 days, suggesting a possible intersecting effect of aging and IR-specific processes. Considering these findings, it is possible accelerated atherosclerosis in IR groups may contribute to altered hemodynamics and the development of LV remodeling. More comprehencive vascular studies are ongoing.

The echocardiography data suggests that LV function may be recovered between 28- and 365-days post-IR, but chronic IR-induced changes with aging may introduce a point of subsequent LV functional decline at 440 days. There was no significant difference in LV structure and function at 660 days, though more considerable inter-animal variability is observed within groups. However, owing to limited animal survival at this time, it is challenging to extrapolate whether any actual CVD endpoints were met when animals are approaching the end of life. Also, increased mortality was seen in irradiated groups at 660 days introducing a possible survivor bias where the healthier animals preferentially survived to this time point. Senescence-associated secretory phenotype (SASP) markers hold the key to unraveling the intricate interplay between aging, IR, and the molecular mechanisms underlying these processes. By assessing SASP markers in our future studies, we aim to not only differentiate between age-related and IR-related effects but also to gain deeper insights into the underlying pathways involved. This comprehensive approach will significantly enhance our understanding of how these factors interact and contribute to the observed outcomes in our long-term study. This may have masked the structural and functional changes at this time point. Since significant decline was seen at 14, 28, and 440 days these time points were focused on to model dose responses in order to look for possible thresholds and calculate RBE and RER. Similar to the previous work with C57BL/6J mice, no indication of a threshold was seen, and RBE were only defined over a relatively narrow dose range. This suggests the use of RER instead of RBE for future risk modeling.

Interestingly, we previously showed that irradiated C57BL/6J mice at early and/or intermediate time points (365 and 440 days) did not reveal alterations in LV function, until 660 days following simGCRsim- or γ-IR exposure, suggesting that the underlying genetic background of mice in this study (i.e., knockout of ApoE) may possibly contribute to varied IR responses. Therefore, it is critical to consider individual disease susceptibility when considering the composite effect of IR exposure to both acute- and long-term CVD risk development.

Although not assessed in this study, it is critical also to consider the effects of sex on radiation-induced CVD. Even within the female sex, there are considerable differences in CVD in peri- and post-menopausal women attributed to a decline in sex steroid hormones (SSH), which are critical determinants of CVD sex differences. In particular, the cardioprotective effects of estrogen and estrogen receptors (ER) have been well studied and have extensive applications as previously reviewed (Mendelsohn and Karas, 2005; Regitz-Zagrosek and Gebhard, 2023), including rapid simulation of vascular endothelial nitric oxide synthase and vascular dilatation, cardiospecific coregulators of ERs, such as steroid receptor coactivator 3 (SRC3) facilitating ER-dependent vasoprotective effects following injury, or the potential prosurival effects of estradiol-ER on cardiomyocytes in preserving myocardial mass with age in females. Outside of SSH related differences in vascular biology, sex differences in electrophysiology have also been noted (Ehdaie et al., 2018). Work from our team is currently underway to assess the sex differences that may arise following IR exposure in longitudinal studies employing both wild-type and ApoE null female mice across their lifespan.

Overall, we showed that LV systolic function is impaired 28 days post-IR with simGCRsim- or γ-IR and is associated with increased LV wall thickness and evidence of hemodynamic stress in ApoE null male mice. Alterations in calcium handling may be a shared pathway associated with LV dysfunction in both IR groups; however, IR-specific effects may be observed regarding the predominant mechanisms driving cardiac remodeling (i.e., fibrosis *versus* hypertrophy). Further work is needed to determine whether there are sex-specific effects associated with IR exposure, in addition to assessing additional cardiovascular endpoints, such as atherosclerosis. While ApoE null male mice did not exhibit detectably increased dysfunction in late stages post-IR (365, 440, and 660 days), there was considerable individual variability and the possibility of a survivor bias that may have been partially masking an effect. Also, the possibility of diastolic dysfunction cannot be excluded. Additionally, the cumulative effects of additional space travel stressors, such as microgravity, must be considered concerning the chronicity and severity of CVD phenotypes that may be observed and thus require further investigation.

## Figures and Tables

**Table:** 

**AIC**	Akaike Information Criterion
***ANP***	Atrial natriuretic peptide
**ApoE**	Apolipoprotein E
***ßMHC***	Beta-myosin heavy chain
**BLAF**	Brookhaven Laboratory Animal Facility
**BMD**	Benchmark Doses
**BNL**	Brookhaven National Laboratory
***BNP***	Brain natriuretic peptide
**CO**	Cardiac output
***Col1a1***	type I fibrillar collagen
***Col3a1***	type III fibrillar collagen
**CVD**	Cardiovascular disease
**DWS**	Diastolic wall strain
**EDV**	End-diastolic volume
**EPA**	Environmental Protection Agency
**ESV**	End-systolic volume
**Freq. Exp. Deg. 4**	Frequentist Exponential degree 4
***Gal-3***	Galectin-3
**GCR**	Galactic cosmic rays
**GE**	General Electric Company
**HF**	Heart failure
**HFpEF**	Heart failure with a preserved ejection fraction
**HRP**	Human Research Program
**HZE**	High charge (H), atomic number (Z), and energy (E)
**IR**	Ionizing radiation
**IVSd**	Intraventricular septal thickness
**LET**	Linear energy transfer
**LV**	Left ventricular/ventricle
**LVEDd**	LV end-diastolic diameter
**LVEF**	LV ejection fraction
**LVFS**	LV fractional shortening
**LVIDd**	LV internal diameter at end diastole
**LVIDs**	LV internal diameter at end-systole
**LVPWd**	LV posterior wall thickness at end-diastole
**LVPWs**	LV posterior wall thickness at end-systole
***Mcp1***	Monocyte chemoattractant protein
***Mmp9***	Matrix metallopeptidase 9
**NASA**	National Aeronautics and Space Administration
**NCX**	Sodium-calcium exchanger
**ND**	Normal diet
**NSRL**	NASA Space Radiation Laboratory
**PWthd**	Posterior wall thickness at end-diastole
**RBE**	Relative biological effectiveness
**RER**	Radiation effects ratio
**RICVD**	Radiation-induces CVD
**RT**	Radiation therapy
**SEP**	Solar energetic particles
***Serca2a***	Sarcoplasmic reticulum Ca^2+^-ATPases
**simGCRsim**	Simplified galactic cosmic ray simulation
**SV**	Stroke volume
***Tgfβ1***	Transforming growth factor-β1
***Tnfα***	Tumor necrosis factor alpha
**WD**	Western diet

## Data Availability

The raw data supporting the conclusion of this article will be made available by the authors, without undue reservation.

## References

[B1] Andreassi M. G. Piccaluga E. Gargani L. Sabatino L. Borghini A. Faita F. (2015). Subclinical carotid atherosclerosis and early vascular aging from long-term low-dose ionizing radiation exposure: a genetic, telomere, and vascular ultrasound study in cardiac catheterization laboratory staff. JACC Cardiovasc Interv. 8 (4), 616–627. 10.1016/j.jcin.2014.12.233 25907089

[B2] Armoundas A. A. Rose J. Aggarwal R. Stuyvers B. D. O'Rourke B. Kass D. A. (2007). Cellular and molecular determinants of altered Ca2+ handling in the failing rabbit heart: primary defects in SR Ca2+ uptake and release mechanisms. Am. J. Physiol. Heart Circ. Physiol. 292 (3), H1607–H1618. 10.1152/ajpheart.00525.2006 17122195 PMC2711877

[B3] Attwood J. T. Yung R. L. Richardson B. C. (2002). DNA methylation and the regulation of gene transcription. Cell Mol. Life Sci. 59 (2), 241–257. 10.1007/s00018-002-8420-z 11915942 PMC11146104

[B4] Barcellos-Hoff M. H. Mao J. H. (2016). HZE radiation non-targeted effects on the microenvironment that mediate mammary carcinogenesis. Front. Oncol. 6, 57. 10.3389/fonc.2016.00057 27014632 PMC4786544

[B5] Berridge M. J. Bootman M. D. Roderick H. L. (2003). Calcium signalling: dynamics, homeostasis and remodelling. Nat. Rev. Mol. Cell Biol. 4 (7), 517–529. 10.1038/nrm1155 12838335

[B6] Berrington A. Darby S. C. Weiss H. A. Doll R. (2001). 100 years of observation on British radiologists: mortality from cancer and other causes 1897-1997. Br. J. Radiol. 74 (882), 507–519. 10.1259/bjr.74.882.740507 11459730

[B7] Bozkurt B. Aguilar D. Deswal A. Dunbar S. B. Francis G. S. Horwich T. (2016). Contributory risk and management of comorbidities of hypertension, obesity, diabetes mellitus, hyperlipidemia, and metabolic syndrome in chronic heart failure: a scientific statement from the American heart association. Circulation 134 (23), e535–e578. 10.1161/CIR.0000000000000450 27799274

[B8] Brojakowska A. Jackson C. J. Bisserier M. Khlgatian M. K. Grano C. Blattnig S. R. (2023). Lifetime evaluation of left ventricular structure and function in male C57BL/6J mice after gamma and space-type radiation exposure. Int. J. Mol. Sci. 24 (6), 5451. 10.3390/ijms24065451 36982525 PMC10049327

[B9] Bruch C. Gradaus R. Gunia S. Breithardt G. Wichter T. (2003). Doppler tissue analysis of mitral annular velocities: evidence for systolic abnormalities in patients with diastolic heart failure. J. Am. Soc. Echocardiogr. 16 (10), 1031–1036. 10.1016/S0894-7317(03)00634-5 14566295

[B10] de Boer R. A. Voors A. A. Muntendam P. van Gilst W. H. van Veldhuisen D. J. (2009). Galectin-3: a novel mediator of heart failure development and progression. Eur. J. Heart Fail 11 (9), 811–817. 10.1093/eurjhf/hfp097 19648160

[B11] Dong R. Zhang M. Hu Q. Zheng S. Soh A. Zheng Y. (2018). Galectin-3 as a novel biomarker for disease diagnosis and a target for therapy (Review). Int. J. Mol. Med. 41 (2), 599–614. 10.3892/ijmm.2017.3311 29207027 PMC5752178

[B12] Ehdaie A. Cingolani E. Shehata M. Wang X. Curtis A. B. Chugh S. S. (2018). Sex differences in cardiac arrhythmias: clinical and research implications. Circ. Arrhythm. Electrophysiol. 11 (3), e005680. 10.1161/CIRCEP.117.005680 29874167

[B13] Ertl G. Frantz S. (2005). Healing after myocardial infarction. Cardiovasc Res. 66 (1), 22–32. 10.1016/j.cardiores.2005.01.011 15769445

[B14] Flesch M. Schwinger R. H. Schiffer F. Frank K. Sudkamp M. Kuhn-Regnier F. (1996). Evidence for functional relevance of an enhanced expression of the Na(+)-Ca2+ exchanger in failing human myocardium. Circulation 94 (5), 992–1002. 10.1161/01.cir.94.5.992 8790037

[B15] Gaggin H. K. Januzzi J. L. Jr. (2013). Biomarkers and diagnostics in heart failure. Biochim. Biophys. Acta 1832 (12), 2442–2450. 10.1016/j.bbadis.2012.12.014 23313577

[B16] Gardin J. M. Siri F. M. Kitsis R. N. Edwards J. G. Leinwand L. A. (1995). Echocardiographic assessment of left ventricular mass and systolic function in mice. Circ. Res. 76 (5), 907–914. 10.1161/01.res.76.5.907 7729009

[B17] Gehlken C. Suthahar N. Meijers W. C. de Boer R. A. (2018). Galectin-3 in heart failure: an update of the last 3 years. Heart Fail Clin. 14 (1), 75–92. 10.1016/j.hfc.2017.08.009 29153203

[B18] Grube E. Silber S. Hauptmann K. E. Mueller R. Buellesfeld L. Gerckens U. (2003). TAXUS I: six- and twelve-month results from a randomized, double-blind trial on a slow-release paclitaxel-eluting stent for *de novo* coronary lesions. Circulation 107 (1), 38–42. 10.1161/01.cir.0000047700.58683.a1 12515740

[B19] Gupta M. P. (2007). Factors controlling cardiac myosin-isoform shift during hypertrophy and heart failure. J. Mol. Cell Cardiol. 43 (4), 388–403. 10.1016/j.yjmcc.2007.07.045 17720186 PMC2701247

[B20] Heidenreich P. A. Hancock S. L. Lee B. K. Mariscal C. S. Schnittger I. (2003). Asymptomatic cardiac disease following mediastinal irradiation. J. Am. Coll. Cardiol. 42 (4), 743–749. 10.1016/s0735-1097(03)00759-9 12932613

[B21] Homburger J. R. Green E. M. Caleshu C. Sunitha M. S. Taylor R. E. Ruppel K. M. (2016). Multidimensional structure-function relationships in human beta-cardiac myosin from population-scale genetic variation. Proc. Natl. Acad. Sci. U. S. A. 113 (24), 6701–6706. 10.1073/pnas.1606950113 27247418 PMC4914177

[B22] Hooning M. J. Botma A. Aleman B. M. Baaijens M. H. Bartelink H. Klijn J. G. (2007). Long-term risk of cardiovascular disease in 10-year survivors of breast cancer. J. Natl. Cancer Inst. 99 (5), 365–375. 10.1093/jnci/djk064 17341728

[B23] Kamisago M. Schmitt J. P. McNamara D. Seidman C. Seidman J. G. (2006). Sarcomere protein gene mutations and inherited heart disease: a beta-cardiac myosin heavy chain mutation causing endocardial fibroelastosis and heart failure. Novartis Found. Symp. 274, 176–189; discussion 189-195, 272-276. discussion 89-95, 272-6. 17019812

[B24] Koutroumpakis E. Deswal A. Yusuf S. W. Abe J. I. Nead K. T. Potter A. S. (2022). Radiation-induced cardiovascular disease: mechanisms, prevention, and treatment. Curr. Oncol. Rep. 24 (5), 543–553. 10.1007/s11912-022-01238-8 35192118

[B25] Landim-Vieira M. Childers M. C. Wacker A. L. Garcia M. R. He H. Singh R. (2022). Post-translational modification patterns on beta-myosin heavy chain are altered in ischemic and nonischemic human hearts. Elife 11, e74919. 10.7554/eLife.74919 35502901 PMC9122498

[B26] Li V. W. Cheuk D. K. Cheng F. W. Yang J. Y. Yau J. P. Ho K. K. (2017). Myocardial stiffness as assessed by diastolic wall strain in adult survivors of childhood leukaemias with preserved left ventricular ejection fraction. Eur. Heart J. Cardiovasc Imaging 18 (4), 451–458. 10.1093/ehjci/jew098 27166023

[B27] Li Y. Koshizaki N. Shimizu Y. Li L. Gao S. Sasaki T. (2009). Unconventional lithography for hierarchical micro-/nanostructure arrays with well-aligned 1D crystalline nanostructures: design and creation based on the colloidal monolayer. ACS Appl. Mater Interfaces 1 (11), 2580–2585. 10.1021/am900513m 20356130

[B28] Little M. P. Azizova T. V. Richardson D. B. Tapio S. Bernier M. O. Kreuzer M. (2023). Ionising radiation and cardiovascular disease: systematic review and meta-analysis. BMJ 380, e072924. 10.1136/bmj-2022-072924 36889791 PMC10535030

[B29] Lo Sasso G. Schlage W. K. Boue S. Veljkovic E. Peitsch M. C. Hoeng J. (2016). The Apoe(-/-) mouse model: a suitable model to study cardiovascular and respiratory diseases in the context of cigarette smoke exposure and harm reduction. J. Transl. Med. 14 (1), 146. 10.1186/s12967-016-0901-1 27207171 PMC4875735

[B30] Mendelsohn M. E. Karas R. H. (2005). Molecular and cellular basis of cardiovascular gender differences. Science 308 (5728), 1583–1587. 10.1126/science.1112062 15947175

[B31] Nakashima Y. Plump A. S. Raines E. W. Breslow J. L. Ross R. (1994). Apoe-deficient mice develop lesions of all phases of atherosclerosis throughout the arterial tree. Arteriosclerosis Thrombosis Vasc. Biol. 14, 133–140. 10.1161/01.atv.14.1.133 8274468

[B32] National Library of Medicine (2002). Effect of intensive therapy on the microvascular complications of type 1 diabetes mellitus. Jama 287 (19), 2563–2569. 10.1001/jama.287.19.2563 12020338 PMC2622728

[B33] Nelson G. A. (2016). Space radiation and human exposures, A primer. Radiat. Res. 185 (4), 349–358. 10.1667/RR14311.1 27018778

[B34] Nielsen K. M. Offersen B. V. Nielsen H. M. Vaage-Nilsen M. Yusuf S. W. (2017). Short and long term radiation induced cardiovascular disease in patients with cancer. Clin. Cardiol. 40 (4), 255–261. 10.1002/clc.22634 28139844 PMC6589645

[B35] Ohtani T. Mohammed S. F. Yamamoto K. Dunlay S. M. Weston S. A. Sakata Y. (2012). Diastolic stiffness as assessed by diastolic wall strain is associated with adverse remodelling and poor outcomes in heart failure with preserved ejection fraction. Eur. Heart J. 33 (14), 1742–1749. 10.1093/eurheartj/ehs135 22645191 PMC3530390

[B36] Paigen B. Holmes P. A. Mitchell D. Albee D. (1987). Comparison of atherosclerotic lesions and HDL-lipid levels in male, female, and testosterone-treated female mice from strains C57BL/6, BALB/c, and C3H. Atherosclerosis 64 (2-3), 215–221. 10.1016/0021-9150(87)90249-8 3606719

[B37] Ramadan S. S. Sridharan V. Koturbash I. Miousse I. R. Hauer-Jensen M. Nelson G. A. (2016). A priming dose of protons alters the early cardiac cellular and molecular response to (56)Fe irradiation. Life Sci. Space Res. (Amst). 8, 8–13. 10.1016/j.lssr.2015.12.001 26948008 PMC4782196

[B38] Reginauld S. H. Cannone V. Iyer S. Scott C. Bailey K. Schaefer J. (2019). Differential regulation of ANP and BNP in acute decompensated heart failure: deficiency of ANP. JACC Heart Fail 7 (10), 891–898. 10.1016/j.jchf.2019.05.012 31521687 PMC6778016

[B39] Regitz-Zagrosek V. Gebhard C. (2023). Gender medicine: effects of sex and gender on cardiovascular disease manifestation and outcomes. Nat. Rev. Cardiol. 20 (4), 236–247. 10.1038/s41569-022-00797-4 36316574 PMC9628527

[B40] Reinders J. G. Heijmen B. J. Olofsen-van Acht M. J. van Putten W. L. Levendag P. C. (1999). Ischemic heart disease after mantlefield irradiation for Hodgkin's disease in long-term follow-up. Radiother. Oncol. 51 (1), 35–42. 10.1016/s0167-8140(99)00026-2 10386715

[B41] Sag C. M. Wolff H. A. Neumann K. Opiela M. K. Zhang J. Steuer F. (2013). Ionizing radiation regulates cardiac Ca handling via increased ROS and activated CaMKII. Basic Res. Cardiol. 108 (6), 385. 10.1007/s00395-013-0385-6 24068185 PMC3898380

[B42] Seawright J. W. Sridharan V. Landes R. D. Cao M. Singh P. Koturbash I. (2019). Effects of low-dose oxygen ions and protons on cardiac function and structure in male C57BL/6J mice. Life Sci. Space Res. (Amst) 20, 72–84. 10.1016/j.lssr.2019.01.003 30797436 PMC6391741

[B43] Selvaraj S. Aguilar F. G. Martinez E. E. Beussink L. Kim K. Y. Peng J. (2014). Diastolic wall strain: a simple marker of abnormal cardiac mechanics. Cardiovasc Ultrasound 12, 40. 10.1186/1476-7120-12-40 25277882 PMC4197332

[B44] Shuryak I. Fornace A. J. Jr. Datta K. Suman S. Kumar S. Sachs R. K. (2017). Scaling human cancer risks from low LET to high LET when dose-effect relationships are complex. Radiat. Res. 187 (4), 476–482. 10.1667/RR009CC.1 28218889

[B45] Simmonds S. J. Cuijpers I. Heymans S. Jones E. A. V. (2020). Cellular and molecular differences between HFpEF and HFrEF: a step ahead in an improved pathological understanding. Cells 9 (1), 242. 10.3390/cells9010242 31963679 PMC7016826

[B46] Simonsen L. C. Slaba T. C. Guida P. Rusek A. (2020). NASA's first ground-based Galactic Cosmic Ray Simulator: enabling a new era in space radiobiology research. PLoS Biol. 18 (5), e3000669. 10.1371/journal.pbio.3000669 32428004 PMC7236977

[B47] Studer R. Reinecke H. Bilger J. Eschenhagen T. Bohm M. Hasenfuss G. (1994). Gene expression of the cardiac Na(+)-Ca2+ exchanger in end-stage human heart failure. Circ. Res. 75 (3), 443–453. 10.1161/01.res.75.3.443 8062418

[B48] van Dijk K. W. Hofker M. H. Havekes L. M. (1999). Dissection of the complex role of apolipoprotein E in lipoprotein metabolism and atherosclerosis using mouse models. Curr. Atheroscler. Rep. 1 (2), 101–107. 10.1007/s11883-999-0005-y 11122698

[B49] Vedin O. Lam C. S. P. Koh A. S. Benson L. Teng T. H. K. Tay W. T. (2017). Significance of ischemic heart disease in patients with heart failure and preserved, midrange, and reduced ejection fraction: a nationwide cohort study. Circ. Heart Fail 10 (6), e003875. 10.1161/CIRCHEARTFAILURE.117.003875 28615366

[B50] Wang X. Palaskas N. L. Yusuf S. W. Abe J. I. Lopez-Mattei J. Banchs J. (2020). Incidence and onset of severe cardiac events after radiotherapy for esophageal cancer. J. Thorac. Oncol. 15 (10), 1682–1690. 10.1016/j.jtho.2020.06.014 32599073 PMC9398884

[B51] Weintraub N. L. Jones W. K. Manka D. (2010). Understanding radiation-induced vascular disease. J. Am. Coll. Cardiol. 55 (12), 1237–1239. 10.1016/j.jacc.2009.11.053 20298931 PMC3807611

[B52] Yan X. Sasi S. P. Gee H. Lee J. Yang Y. Mehrzad R. (2014). Cardiovascular risks associated with low dose ionizing particle radiation. PLoS One 9 (10), e110269. 10.1371/journal.pone.0110269 25337914 PMC4206415

[B53] Yue D. T. Marban E. Wier W. G. (1986). Relationship between force and intracellular [Ca2+] in tetanized mammalian heart muscle. J. Gen. Physiol. 87 (2), 223–242. 10.1085/jgp.87.2.223 2419483 PMC2217602

