# Supplementary material for: Lifetime evaluation of left ventricular structure and function in male ApoE null mice after gamma and space-type radiation exposure
Source: Front Physiol. 2023 Nov 20;14:1292033. doi: 10.3389/fphys.2023.1292033 (PMC10694360; doi:10.3389/fphys.2023.1292033)
Supplement: Supplementary file 1 [file Table1.DOCX]

|  |  |  | **Time post Irradiation** | | | | |  |
| --- | --- | --- | --- | --- | --- | --- | --- | --- |
|  | **Dose (cGy)** | **Diet** | **14 days** | **28 days** | **365 days** | **440 days** | **660 days** | **Total #/**  **Timepoint** |
| **No Irradiation** | **0** | **Normal Diet** | #5 | #5 | #20 | #20 | #40 | #90 |
|  | **0** | **Western Diet** | #5 | #5 | #20 | #20 | #40 | #90 |
| **simGCRsim** | **50** | **Normal Diet** | #5 | #5 | #20 | #20 | #40 | #90 |
|  | **100** | **Normal Diet** | #5 | #5 | #20 | #20 | #40 | #90 |
|  | **150** | **Normal Diet** | #5 | #5 | #20 | #20 | #40 | #90 |
| **Gamma** | **100** | **Normal Diet** | #5 | #5 | #20 | #20 | #40 | #90 |
|  | **200** | **Normal Diet** | #5 | #5 | #20 | #20 | #40 | #90 |
|  | **400** | **Normal Diet** | #5 | #5 | #20 | #20 | #40 | #90 |
|  | **Total Number Timepoint** | | #40 | #40 | #160 | #160 | #320 | #720 |

**Supplementary Table 1.** Number of animals for experiment per treatment condition and time point.

|  | **No-IR** | | **simGCRsim-IR** | | | **γ-IR** | | |
| --- | --- | --- | --- | --- | --- | --- | --- | --- |
| **Pathology** | **ND-fed** | **WD-fed** | **50 cGy** | **100 cGy** | **150 cGy** | **100 cGy** | **200 cGy** | **400 cGy** |
| ***Myocardial injury/vascular injury*** | | | | | | | | |
| Marked, multifocal extensive atherosclerosis | 2 |  | 4 | 2 | 2 | 4 | 4 | 3 |
| Mild multifocal myocardial fibrosis, myofiber disarray and myofiber hypertrophy |  |  | 2 |  |  | 2 | 4 | 3 |
| ***Sepsis and/or infectious disease/inflammation*** | | | | | | | | |
| Acute interstitial pneumonia with intravascular bacteria |  |  | 1 | 1 |  | 1 |  |  |
| Severe, red pulp extramedullary hematopoiesis |  |  |  | 1 |  |  |  | 1 |
| ***Cancer*** | | | | | | | | |
| Metastatic Lymphoma |  | 1 |  | 2 | 2 | 2 |  | 2 |
| Alveolar/bronchiolar carcinoma |  |  | 1 |  |  |  |  |  |
| Hepatocellular carcinoma |  |  |  | 2 |  |  |  |  |
| Histiocytic sarcoma |  |  |  |  | 1 |  |  |  |
| Hemangiosarcoma |  |  |  |  |  |  | 2 |  |
| ***Possible toxic injury*** | | | | | | | | |
| Severe diffuse vacuolar degeneration in liver |  | 1 |  |  |  | 1 |  |  |
| Liver infarct |  |  |  |  |  | 1 |  |  |
| Splenic infarct |  |  |  |  |  | 1 |  |  |
| ***Inflammation*** |  |  |  |  |  |  |  |  |
| Mild to moderate multifocal perivascular lymphoid aggregates in liver |  |  |  |  |  |  | 1 |  |
| Moderate, multifocal chronic glomerulonephropathy |  |  |  |  |  | 1 |  |  |

**Supplementary Table 2.** Pathology results for animals necropsied.
